# Rare Taxa Drive the Response of Soil Fungal Guilds to Soil Salinization in the Taklamakan Desert

**DOI:** 10.3389/fmicb.2022.862245

**Published:** 2022-05-23

**Authors:** Litao Lin, Xin Jing, Manuel Esteban Lucas-Borja, Congcong Shen, Yugang Wang, Wenting Feng

**Affiliations:** ^1^Institute of Agricultural Resources and Regional Planning, Chinese Academy of Agricultural Sciences, Beijing, China; ^2^State Key Laboratory of Desert and Oasis Ecology, Xinjiang Institute of Ecology and Geography, Chinese Academy of Sciences, Urumqi, China; ^3^State Key Laboratory of Grassland Agro-Ecosystems, College of Pastoral Agriculture Science and Technology, Lanzhou University, Lanzhou, China; ^4^Technical School of Agricultural and Forest Engineering (ETSIAM), University of Castilla-La Mancha (UCLM), Albacete, Spain; ^5^State Key Laboratory of Urban and Regional Ecology, Research Center for Eco-Environmental Sciences, Chinese Academy of Sciences, Beijing, China; ^6^University of Chinese Academy of Sciences, Beijing, China; ^7^Fukang Station of Desert Ecology, Chinese Academy of Sciences, Fukang, China

**Keywords:** biodiversity, desert, functional guilds, rare biosphere, soil salinization, saprotrophic fungi, symbiotrophic fungi

## Abstract

Salinization poses great threats to soil fungal communities that would cause the losses of ecosystems services. Soil fungal communities are composed of different functional guilds such as saprotrophic, symbiotrophic, and pathotrophic fungi, and each guild includes many rare taxa and a few abundant taxa. Despite of low abundance, rare taxa may be crucial in determining the responses of entire soil fungal communities to salinization. However, it remains poorly understood how rare taxa mediate the impacts of soil salinization on soil fungal community structure. Here, we took advantage of a salinity gradient in a desert ecosystem ranging from 0.60 to 31.09 g kg^−1^ that was created by a 12-year saline-water irrigation and assessed how the rare vs. abundant taxa of soil saprotrophic, symbiotrophic, and pathotrophic fungi respond to soil salinization through changes in the community biodiversity and composition. We found that the rare taxa of soil saprotrophic, symbiotrophic, and pathographic fungi were more sensitive to changes in soil salinity compared to the abundant taxa. In addition, the community composition of rare taxa of the saprotrophic and pathotrophic fungi not the symbiotrophic fungi was positively associated with soil salinity change. However, the symbiotrophic fungi showed greater variations in the species richness along the salinity gradient. These findings highlight the importance to differentiate rare taxa in predicting how the biodiversity and functional groups of soil fungal communities respond to soil salinization.

## Introduction

Salinity affects about 1.1 billion hectares of land surface around the world, accounting for 7.4% of global land area (Ivushkin et al., 2019). Soil salinization is becoming a major environmental challenge specifically in drylands due to climate change and/or poor land management, such as saline-water irrigation (Rath and Rousk, 2015). Soil salinization causes osmotic pressure, nutritional imbalance, and ion toxic effects to plants and microorganisms (Mansour, 2000; Munns and Tester, 2008), which threatens ecosystem functions and services (Rath et al., 2016). As one of the most diverse and abundant groups of soil microbiota, soil fungi are important for mitigating the negative impacts of salinization on soil functionality (Ruppel et al., 2013; Frac et al., 2018). For instance, soil fungi use low-quality organic matter in infertile soils (Bardgett, 2005) and show higher resistance to environmental stresses over soil bacteria (Rath et al., 2016; Thiem et al., 2018). Soil fungi display stable network attributes and can maintain high fungal biomass per unit mass of soil organic matter under saline conditions (de Vries et al., 2018; Rath et al., 2019). Therefore, undersatnding the impacts of soil salinization on fungal communities is vital for the conservation of belowground biodiversity in the deteriorating dryland environments.

Soil fungal communities are functionally diverse and can be classified into three major functional guilds based on their trophic modes, such as saprotrophic fungi, pathotrophic fungi, and symbiotrophic fungi (Yang et al., 2016; Frac et al., 2018), which may adapt to soil salinization differently. Saprotrophic fungi decompose litter and transform nutrients and their activities depend on the quality and quantity of soil organic matter (Rath et al., 2019). In contrast, pathotrophic fungi affect disease, pests, and the growth of other organisms (Frac et al., 2018). Symbiotrophic fungi improve plant nutrition by establishing plant-mycorrhizal associations, and their abundance and composition depend on soil nutrient conditions (Moore et al., 2021). Given various functions of soil fungal guilds, treating soil fungal communities a whole without differentiating functional differences may mislead the prediction regarding how the functionality of soil fungal communities responds to salinization. For instance, the relative abundance of saprotrophic fungi increases and that of pathotrophic fungi decreases after ecosystem restoration in saline-alkaline soils (Xu et al., 2021). Although fungal taxonomic groups increase or decrease significantly with salinity changes (Kim et al., 2019; Yang et al., 2020), the responses of functional guilds of soil fungi to salinization are still poorly understood. This hinders our understanding of the mechanisms driving for soil fungal communities in dealing with soil salinization and the cascading effects on ecosystem processes.

Communities of soil fungal guilds are comprised of many rare taxa and a few abundant taxa (Balbuena et al., 2021). Current studies mainly focus on the abundant soil fungi, while little is known about how the rare fungal taxa respond to soil salinization. Although a number of studies report that fungal diversity does not change along salinity gradients (Mohamed and Martiny, 2011; Bharti et al., 2015), increasing evidences show that rare fungal taxa are more sensitive to salinity or other stresses than abundant taxa (Wan et al., 2021). Compared with the abundant taxa, the rare taxa of soil fungal guilds have fewer niches and less clustered phylotypes and can be influenced more by selective pressure and dispersal effects, thus they would be more likely subject to extinction and local environmental variations (Galand et al., 2009; Pascoal et al., 2021; Wan et al., 2021). Evidence from forest and wetland soils show bigger changes in the rare microbial taxa with environmental variations compared to the abundant taxa (Oono et al., 2017; Ji et al., 2020; Wan et al., 2021).

In addition, soil functional fungal guilds have different responses to salinity changes (Xu et al., 2021), which might be induced by the rare taxa. Vanegas et al. (2019) have found that saprotrophic fungi have higher sequence abundance than the symbiotrophic fungi and were expected to be more resistant to salinity changes than the latter. But another study observed that saprotrophic fungi displayed greater variations with salinity changes than symbiotrophic fungi (Bencherif et al., 2015), and that the sequenicng abundance of some rare taxa of saproptrophic fungi was higher than that of the abundant taxa of symbiotrophic fungi (Yang et al., 2019). These findings suggest that the rare taxa of saprotrophic fungi may determine the responses of this functional fungal guild to salinity change. Therefore, we need to elucidate how the rare vs. abundant taxa of functional fungal guilds respond to soil salinization in order to better understand the adaptation mechanisms of soil fungal community in dealing with salinity stress.

**Figure 1:**
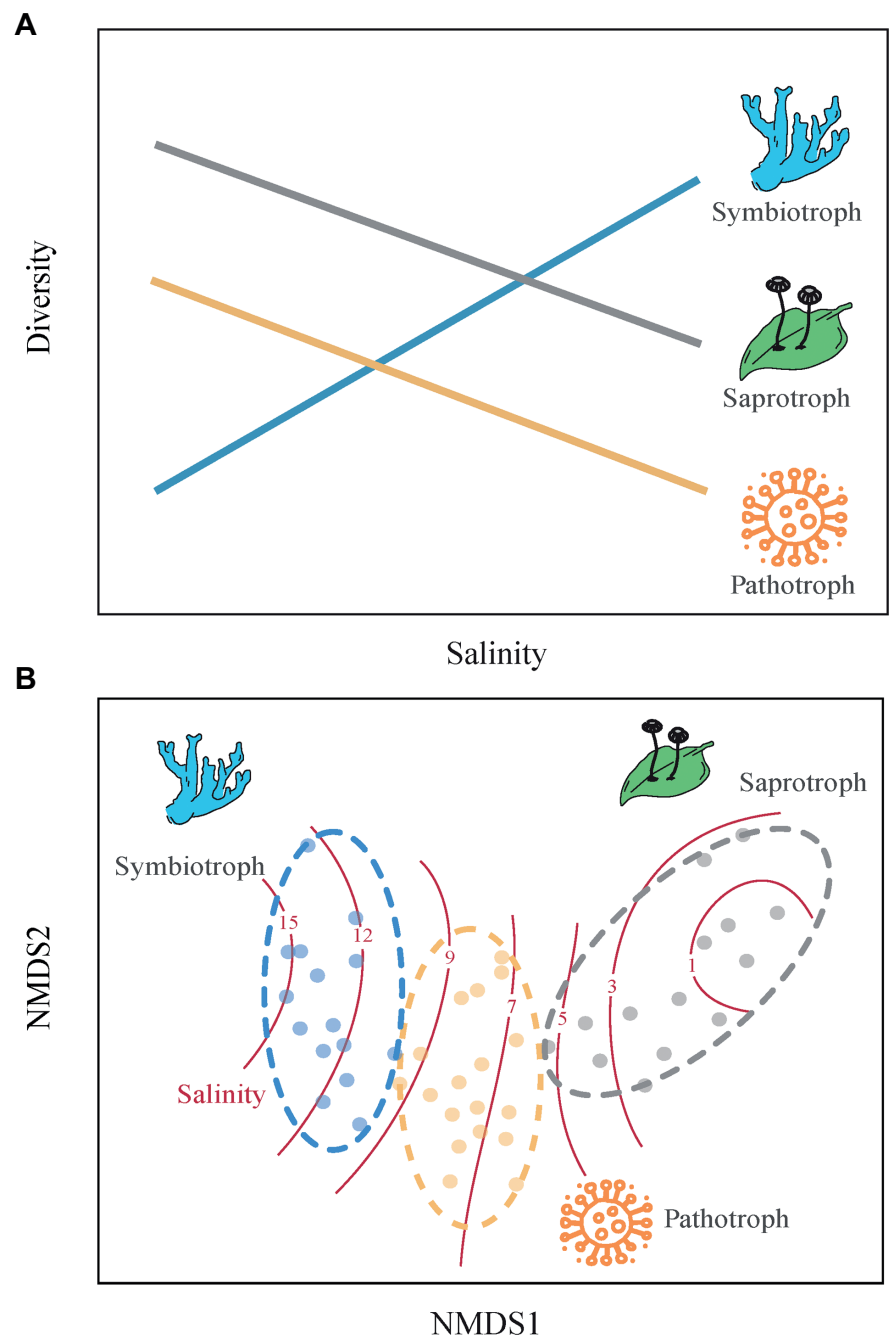
The conceptual framework outlines changes in community structure of three functional guilds of soil fungi along a soil salinity gradient. **(A)** Alpha diversity. We expect that the alpha-diversity of symbiotrophic fungi would increase with soil salinity because symbiotrophic fungi depend highly on host plant at high-salinity sites (Ren et al., 2016), while that of soil saprotrophic and pathotrophic fungi would decrease with soil salinity because high salinity can hinder the activity of soil fungi that mainly use soil organic matter (Rath et al., 2016). **(B)** Community composition. Since soil symbiotrophic, sapraotrophic, and pathotrophic fungi have different niches (Frac et al., 2018), we therefore expect that symbiotrophic fungi occur at higher salinity levels, then do pathotrophic and sapraotrophic fungi.

In this study, we examined the spatial variability in soil fungal community along a soil salinity gradient ranging from 0.60 to 31.09 g kg^−1^ to address two questions: (1) How do the diversity and community composition of three fungal guilds respond to soil salinization? (2) Do the rare fungal taxa determine the response of soil fungal communities to salinization? We predict that the diversity of symbiotrophic fungi increases with soil salinity and that of saprotrophic and pathotrophic fungi decrease along the salinity gradient, because symbiotrophic fungi depends more on plants in high saline soils but the growths of sapraotrophic and pathotrophic fungi are inhibited (Bencherif et al., 2015). In addition, symbiotrophic fungi are expected to occupy more niches and sapraotrophic and pathotrophic fungi occupy less niches with increasing soil salinity (Figure 1). Due to the weaker adaption of microbial rare taxa to environmental changes compared to abundant taxa (Montoya-Ciriaco et al., 2020), we predicted that the rare taxa of soil functional fungal guilds are more sensitive to soil salinization than the abundant taxa.

## Materials and Methods

### Study Site and Sampling

The study sites are in the shrub shelterbelts along the Tarim desert highway, which crosses the Taklamakan desert with a total length of 465 km. The Taklamakan desert has a continental hyper arid climate with mean annual temperature of 2.4°C, mean annual precipitation of 24.6 mm, and mean annual potential evaporation of 3,639 mm. The Taklamakan desert has an average wind speed of 5.6 mph and gale (> 44.7 mph) occurs > 130 days·year^−1^ (Lei et al., 2008). Soils in the Taklamakan desert are aeolian sandy soils with 87.3% sand, 12.4% silt, and 0.3% clay (Zhang et al., 2016). In 2005, the shrub shelterbelts (72 ~ 78 m in width) were constructed to prevent mobile sand dunes from covering the Tarim desert highway by planting drought and salt tolerant species, such as *Haloxylon ammodendron* (C. A. Mey.) Bunge., *Tamarix chinensis* Lour., and *Calligonum mongolicum* Turcz (Li, 2010). Due to the hyper arid environment of the Taklamakan desert, groundwater has been pumped for drip irrigation to water the shelterbelts from March to October regularly (Zhang et al., 2016). The 12-year drip irrigation by using saline groundwater and high evaporation have resulted in soil salinization (Wang et al., 2012), and *H. ammodendron* becomes the dominant plant species in the shelterbelts (Li, 2010; Zhang et al., 2016).

The soil salinity gradient was caused by a 12-year saline-water irrigation in the shrub shelterbelts along the Tarim desert highway. The salinity of groundwater used for irrigation decreases from 30.0 to 2.6 g·L^−1^ from the north to the middle of the Tarim desert highway (Li et al., 2008), and salts accumulate in topsoils along soil profiles due to high evaporation (Supplementary Figure 1). Then, soil salinity genearlly decreases from the north to the middle of the Tarim desert highway and also decreases from surface soils to subsoils. In addition, plants in the shrub shelterbelts are dominated by *Haloxylon ammodendron* (C. A. Mey.) Bunge., which minimizes the influence of plant variation on fungal guilds and provides great opportunities to investigate the impacts of soil salinization on soil fungi in field. Plants in the shrub shelterbelts were arranged in rows, with a spacing of 2 m between rows and a spacing of 1 m between individuals within each row.

In this study, ten sites in the shelterbelts were selected from the north to the middle of the Tarim desert highway (Supplementary Figure 2). At each site, three spots were randomly selected from locations that were within the two rows of shrubs and about 30-50 cm away from a shrub along the shrub shelterbelts as replications. Soils were collected using soil cores (5 cm in diameter) at the depths of 0–10, 10–20, 20–40, 40–60, 60–80, and 80–100 cm. A total of 180 soil samples were collected from the ten sites in early October 2017. Soils were passed through a 2 mm sieve to remove coarse roots and stones with visible roots picked by hand. A subset of soils for DNA extraction were stored at −20°C, and another subset soils were air-dried to determine soil properties. The details of soil properties along soil depth were listed at Supplementary Figure 1.

### Soil Properties Analysis

Soil salinity, electrical conductivity (EC), and pH were assessed in a suspension with the soil to water ratio of 1:5 (w/v). The sum of Na^+^, K^+^, Ca^2+^, Mg^2+^, Cl^−^, ${\mathrm{SO}}_{4}^{2-}$ and ${HCO}_{3}^{-}$ excluded ${CO}_{3}^{2-}$ were calculated as soil salinity. The concentration of Na^+^, K^+^, Ca^2+^, and Mg^2+^ were measured by inductively coupled plasma – optical emission spectrometry (ICP-OES, Optima 6,300 DV, Perkin Elmer, United States). Soil Cl^−^ was measured by the AgNO_3_ titration method, ${\mathrm{SO}}_{4}^{2-}$ by ethylenediaminetetraacetic acid (EDTA) titration method, and ${CO}_{3}^{2-}$ and ${HCO}_{3}^{-}$ by the H_2_SO_4_ titration method. Soil pH and EC were measured by a pH and conductivity meter (Mettler Toledo FiveEasy Plus FE38, Greifensee, Switzerland). Soil organic carbon (SOC) was measured by the K_2_Cr_2_O_7_ oxidation method (Nelson and Sommers, 1982). Soil total nitrogen (STN) was measured by an Auto Kjeldahl Unite model (BUCHI K370, Flawil, Switzerland). Soil total phosphorus (STP) was determined by the Mo-Sb colorimetric method (Yuan and Lavkulich, 1995), and soil available P (avaP) was measured using the sodium bicarbonate extraction with Mo-Sb Anti-spectrophotometer method (Olsen, 1954).

### Molecular Analysis

Total soil DNA was extracted from 0.25 g of each fresh sample using a PowerSoil DNA Kit (Qiagen, Carlsbad, CA, United States). The final DNA quality and content was measured by NanoDrop (Thermo Fisher Scientific, United States). The fungal internal transcribed spacer 1 (ITS1) regions of nuclear ribosomal RNA genes were amplified using modified ITS5 (Forward primer, ITS5, 5′-GGAAGTAAAAGTCGTAACAAGG-3′; White et al., 1990) and ITS2 (reverse primer, ITS2, 5’-GCTGCGTTCTTCATCGATGC-3′; Smith and Peay, 2014). PCR was conducted at a total volume of 50 μl, containing 25 μl Premix Taq polymerase, 1 μl of each primer (10 mmol·L^−1^), 60 ng template DNA, and double-distilled H_2_O to a final reaction volume of 50 μl. The following cycling parameters used were: 94°C for 5 min; 30 cycles at 94°C for 30 s, 52°C for 30 s, and 72°C for 30 s; and a final step at 72°C for 10 min. The PCR products were examined on ethidium bromide-stained 1% agarose gels by electrophoresis and visualized under UV light. Three replicates of the PCR products from one sample were pooled together. The PCR products were purified using the EZNA Gel Extraction Kit (Omega, United States). This DNA mix was then sequenced on Illumina PE250 platform (Illumina, United States).

### Bioinformatics Analyses

The sequences were quality-filtered by using Trimmomatic V0.33^1^ and merged by using FLASH V1.2.11^2^ based on the following criteria: the minimum sequence length ≥ 100 bp (excluding barcode and primer sequences); maximum number of primer or barcode mismatches ≤ 2; and minimum mean quality score ≥ 20 in a window of 50 nt; homopolymer length ≤ 10 bp; homopolymer mismatch ratio ≤ 0.1. After quality filtering, 180 samples yielded a total of 1,597,974 sequences, ranging from 37,009 to 157,855 sequences per sample. The sequences were subjected to *de novo* chimaera detection and clustered into operational taxonomic units (OTUs) at a 97% cutoff sequence similarity by using the USEARCH algorithm. The most abundant sequences were chosen as representative sequences by using UC criteria. OTU sequences were then assigned taxonomies using the RDP classifier method against the UNITE database. All samples yielded a total of 11,289,935 fungal sequences, ranging from 3,699 to 150,718 sequences per sample. Fungal OTU clusters and clusters with <2 reads or < 2 samples were discarded from further processing (Lindahl et al., 2013). Since more sequences per sample will help detect rare taxa, the OTU table in this study was rarefied without replacement to 10,898 reads per sample, which was the 10th smallest number of sequences among all the samples (1 sample from the soil depth of 20–40 cm, 2 samples from 40 to 60 cm, 2 samples from 60 to 80 cm, and 3 samples from 80 to 100 cm) and the smallest number of sequences for the samples from top soil layers. After a standard dilution of fungal sequences set by using the rrarefy command in vegan package (Oksanen et al., 2019), we ultimately obtained 5,422 fungal OTUs. These 5,422 fungal OTUs were blasted against the FUNGuild database (Nguyen et al., 2016). For the fungal OTUs that may have multiple trophic modes, they were classified to fungal functional guilds according to their first trophic mode (Brinkmann et al., 2019). We got 722 saprotrophic fungal OTUs, 742 pathotrophic fungal OTUs, and 177 symbiotrophic fungal OTUs in total. The relative abundance of these three fungal guilds was listed at Supplementary Table 1 and Supplementary Figure 3. Further analyses were conducted for these three fungal guilds. The representative sequences of fungal OTUs were submitted to the European Nucleotide Archive (ENA), which is an archive with a history of 30 years and provides free and unlimited access to the annotated sequences of DNA and RNA, at European Molecular Biology Laboratory - European Bioinformatics Institute (EMBL-EBI) under accession number PRJEB46878.^3^

### Statistical Analyses

One of the goals of this study is to compare the rare with the abundant taxa within each fungal guild. Although there are multiple ways to estimate the diversity of rare and abundant taxa, such as relative abundance, frequency, and weights assignment (Supplementary Table 2), the metrics of alpha diversity and community composition were used to assess the changes of rare and abundant taxa of three fungal guilds along the salinity gradient. Because the relative abundance of symbiotrophic fungi was much lower than that of the other two guilds in the studied ecosystem – Taklamakan desert, using the relative abundance approach (e.g., 0.01% or 0.1% threshold; Liu et al., 2015; Lynch and Neufeld, 2015) would play down the population of abundant symbiotrophic fungi, while using the approach of alpha-diversity metrics can adjust the three fungal guilds to an equal level. The approach of alpha-diversity metrics has been used in many studies defined as a flexible tool to investigate the relative contributions of rare vs. common taxa to changes in community diversity as well as the responses of rare and abundant taxa to environment changes (Gossner et al., 2016; Xu et al., 2020). Besides, we examined the rare and abundant taxa of fungal functional guilds by using the 0.01% threshold method, and found that results analyzed by using these two methods were similar for the saprotrophic and pathogenic fungal guilds (Supplementary Tables 12–15).

**Figure 2:**
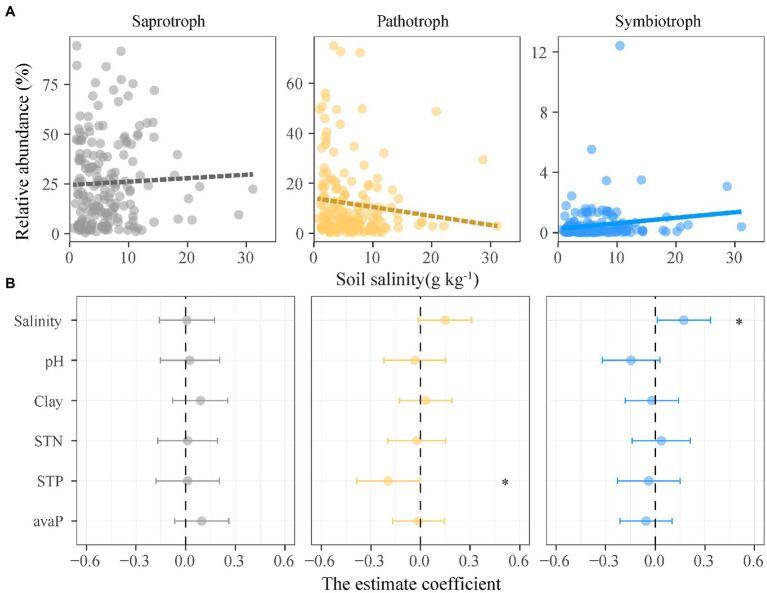
Relationships between the relative abundance of functional guilds of soil fungi and soil salinity and properties. **(A)** Lines are derived from the linear mixed effects models, and solid and dashed lines denote significant and insignificant regression coefficients, respectively. **(B)** Points denote regression coefficients and error bars denote 95% confidence intervals. ^*^p < 0.05. Abbreviations: Salinity, soil salinity; STN, soil total nitrogen; Clay, soil clay content; STP, soil total phosphorus; avaP, soil available phosphorus.

**Figure 3:**
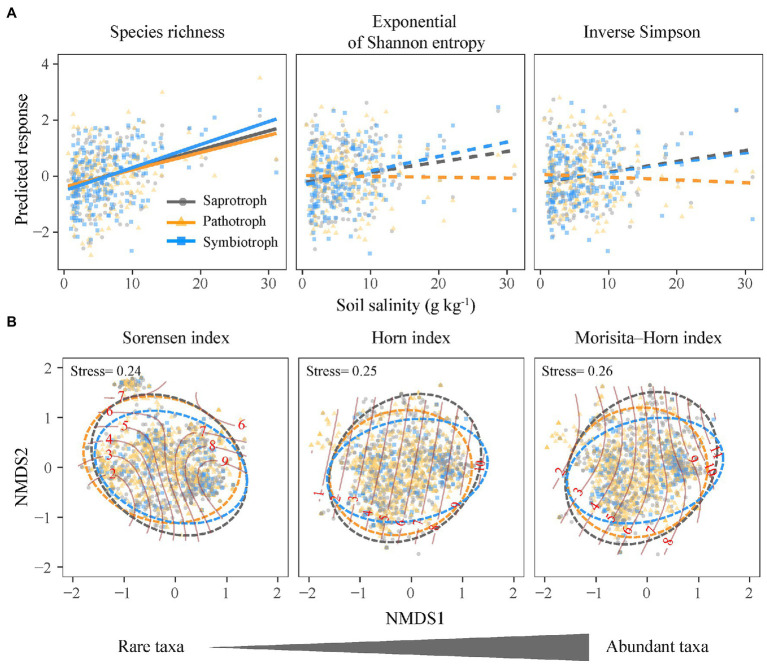
Relationships between the community structure of functional guilds of soil fungi and soil salinity. **(A)** Alpha diversity; lines are derived from the linear mixed effects models and solid and dashed lines denote significant and insignificant regression coefficients, respectively. **(B)** Community composition; solid lines denote salinity gradients and dashed lines denote 0.95 confidence ellipses.

**Figure 4:**
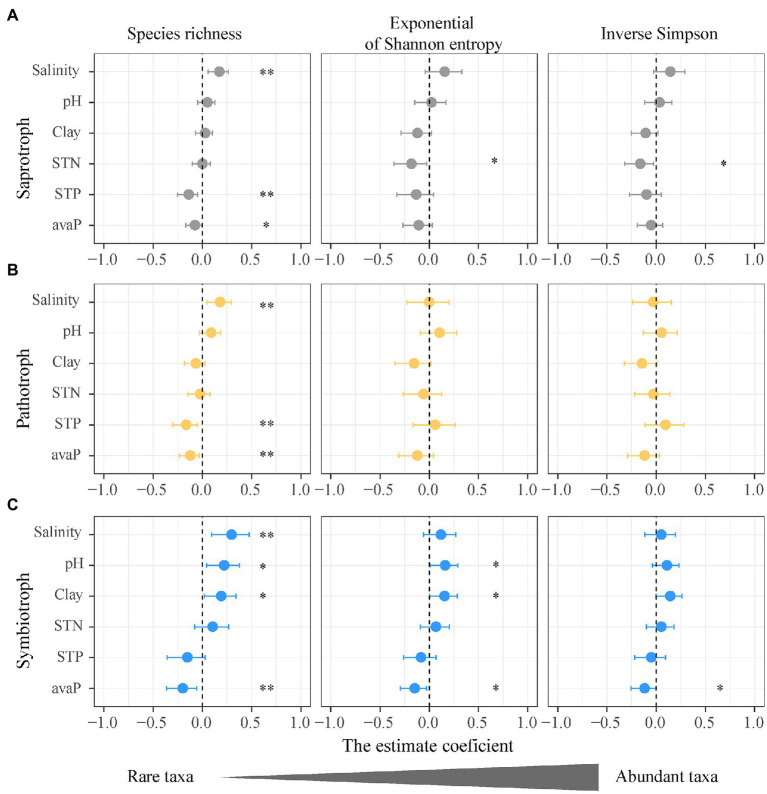
Impacts of soil salinity and properties on the alpha diversity of functional guilds of soil fungi. **(A)** Saprotrophic fungi; **(B)** pathotrophic fungi; **(C)** symbiotrophic fungi. Points denote regression coefficients and error bars denote 95% confidence intervals. ^*^p < 0.05; ^**^p < 0.01. Abbreviations: Salinity, soil salinity; STN, soil total nitrogen; Clay, soil clay content; STP, soil total phosphorus; avaP, soil available phosphorus.

As for the approach of alpha-diversity metrics, the estimate of species richness (*q* = 0) is weighted equally for the rare and abundant species and used to represent the contributions of rare taxa to community diversity. The estimate of the exponential of Shannon entropy (*q* = 1) is weighted in proportion to species frequency in a community and interpreted as the effective number of species. The estimate of inverse Simpson index (*q* = 2) gives more weights to abundant taxa than to rare taxa. It is interpreted as the effective number of very abundant species. The inverse Simpson (*q* = 2) and exponential of Shannon entropy (*q* = 1) indices are used to represent the contributions of abundant taxa to community diversity. We calculated these alpha-diversity indices by using vegan package 2.5–4 (Oksanen et al., 2019).


$${}^{q}H={\left(\overset{S}{\underset{i=1}{\sum }}P_{i}\right)}^{1/\left(1-q\right)}$$

where ^q^*H* is the alpha diversity; *S* is the number of species in the community; *P_i_* represents the relative abundance of the *i*th species in the community; *q* is the order of Hill number. The *q* values determine the sensitivity of alpha-diversity indices to rare and abundant species (Hill, 1973), such as *q* = 0 for species richness, *q* = 1 for the exponential of Shannon entropy, and *q* = 2 for the inverse Simpson index (Table 1). We also calculated three indices of fungal composition dissimilarity for each fungal guild: Sorensen index, Horn index, and Morisita-Horn index (Jing et al., 2021). The three indices are a function of *q* values, which are disproportionately sensitive to the rare and abundant species (Chao et al., 2014). Specifically, the estimate of Sorensen index (*q* = 0) uses binary data (i.e., the rare and abundant species have equal weights) and characterizes the community composition response of rare taxa to soil salinization. The estimate of Horn index (*q* = 1) uses quantitative data and is weighted in proportion to species frequency. The estimate of Morisita-Horn index (*q* = 2) favors abundant taxa more than rare taxa. The Morisita-Horn (*q* = 2) and Horn (*q* = 1) indices are used to represent the community composition response of abundant taxa to soil salinization (Table 1). We used linear mixed-effects models (LMMs) to examine the effects of soil salinity on the relative abundance and alpha diversity of three fungal guilds. We set soil pH, soil total nitrogen, soil total phosphorus, and available phosphorus as fixed factors to control for the impacts of soil salinity on relative abundance and alpha diversity of fungal guilds. We set soil depth and sampling sites as random effects in the models. We did not use soil organic carbon in the models, because soil organic carbon was highly correlated with soil salinity (*r* = 0.70, *p* < 0.01; Supplementary Figure 4). All the factors in the models were subject to forward selection until VIF < 3. We considered soil depth as a random factor because the relative abundance and alpha diversity of three fungal guilds did not significantly differ across soil depths (Supplementary Figure 3) and the interaction between salinity and soil depth showed no significant influence on the relative abundance (Supplementary Table 3) and alpha diversity (Supplementary Tables 4–6) of three fungal guilds. We used both *t* tests and chi-square tests to examine the significance of variables on the relative abundance of three fungal guilds (Supplementary Table 7). Bivariate regressions and coefficients were used to determine and visualize the effects of soil salinity and soil properties on the relative abundance of fungi (Figure 2). We used both *t* tests and chi-square tests to examine the significance of soil salinity and properties on fungal alpha diversity (Supplementary Tables 8–10). Bivariate regressions and coefficients were used to determine and visualize the effects of soil salinity (Figure 3) and other soil properties (Figure 4) on fungal alpha diversity.

**Table 1:** Summary of alpha and beta diversity indices used in this study.

Indices		Definition	Formula	References
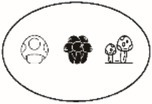	Species richness (*q* = 0)	Number of species	$\overset{S}{\underset{i=1}{\sum }}P_{i}^{0}$	Jost, 2007
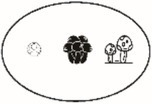	Exponential of Shannon entropy (*q* = 1)	Effective number of typical or abundant species	$\exp\left(-\overset{S}{\underset{i=1}{\sum }}P_{i}\ln P_{i}\right)$	Jost, 2007
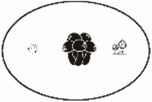	Inverse Simpson (*q* = 2)	Effective number of dominant or very abundant species	$1/\overset{S}{\underset{i=1}{\sum }}P_{i}^{2}$	Jost, 2007
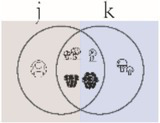	Sorensen index (*q* = 0)	Community dissimilarity using binary data	$\frac{b+c}{2a+b+c}$	Chao et al., 2014
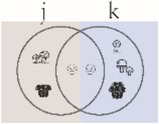	Horn index (*q* = 1)	Community dissimilarity with species weighted in proportion to species abundance	$1-\frac{\sum_{i=1}^{S}\left[P_{ij}\ln\left(1+\frac{P_{ik}}{P_{ij}}\right)+P_{ik}\ln\left(1+\frac{P_{ij}}{P_{ik}}\right)\right]}{2\ln\left(2\right)}$	Chao et al., 2014
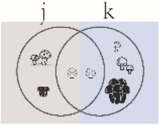	Morisita–Horn index (*q* = 2)	Community dissimilarity with species weighted more to dominant or very abundant species	$1-2\frac{\sum_{i=1}^{S}P_{ij}P_{ik}}{\sum_{i=1}^{S}P_{ij}^{2}+\sum_{i=1}^{S}P_{ik}^{2}}$	Chao et al., 2014

P_i_ = the abundance of the ith species in the assemblage; S = the number of species; P_ij_ or P_ik_ = abundance of the ith species in the jth or kth assemblage; a = the number of species shared in the jth or kth assemblage; b or c = the numbers of unique species in the jth or kth assemblage. Different sizes of fungi in cartoon denote weights during indices calculation.

**Figure 5:**
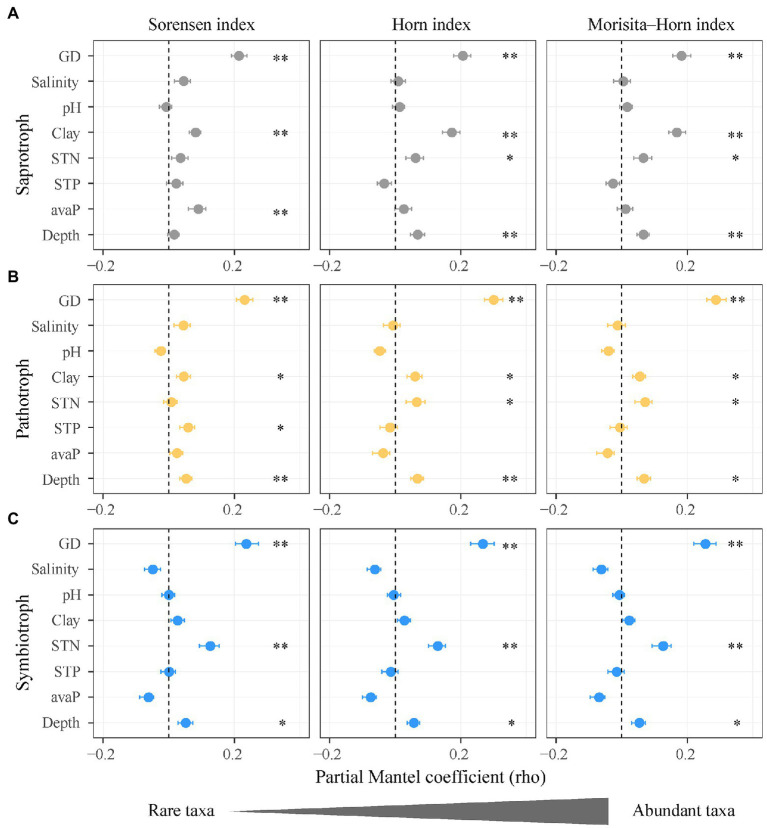
Impacts of soil salinity, geographic distance, and soil properties on the community composition of functional guilds of soil fungi. **(A)** Saprotrophic fungi; **(B)** pathotrophic fungi; **(C)** symbiotrophic fungi. Points denote partial Mantel coefficients and error bars denote 95% confidence intervals. ^*^p < 0.05; ^**^p < 0.01. Abbreviations: GD, geographic distance; Salinity, soil salinity; STN, soil total nitrogen; Clay, soil clay content; STP, soil total phosphorus; avaP, soil available phosphorus; Depth, soil depth.

In addition, the Mantel tests (distance approach) and ordination methods (raw-data approach) have been extensively used in studying community composition variation (beta diversity; Tuomisto and Ruokolainen, 2006). In this study, three indices of beta diversity were used to investigate the rare and abundant fungal taxa variations along the salinity gradient. Therefore, the (partial) Mantel tests were used to examine the relationships between soil fungal composition and geographic distance, soil depth, and environmental factors (Supplementary Table 11). In the Mantel tests, we used the Euclidean distances of zero-mean normalized geographic distances, soil depth, and environmental factors as the fixed factors. We used nonmetric multidimensional scaling (NMDS) that was calculated by metaMDSiter and wascores functions in vegan package to visualize the effect of salinity on niche differentiation among three fungal guilds (Figure 3). We used the bivariate partial Mantel coefficients to visualize the effects of changes in geographic distance and soil properties on community dissimilarity (Figure 5).

## Results

### Relative Abundance of Functional Fungi Along the Salinity Gradient

Saprotrophic, pathotrophic, and symbiotrophic fungal guilds accounted for 25.1, 12.0, and 0.5% of the fungal abundance, respectively (Supplementary Table 1). The unassigned fungal taxa that blasted against FUNGuild database accounted for 62.4% of the fungal abundance. The relative abundance of three fungal guilds showed different patterns along the soil salinity gradient (Figure 2). The relative abundance of symbiotrophic fungi significantly increased with increasing soil salinity, but those of saprotrophic and pathotrophic fungi were not correlated with soil salinity (Figure 2; Supplementary Table 7).

### Alpha Diversity and Composition of Functional Fungi Along the Salinity Gradient

The species richness (*q* = 0) of saprotrophic, pathotrophic, and symbiotrophic fungi all increased with increasing soil salinity (Figure 3A), and the slope of the standardized regressions between symbiotrophic richness and soil salinity (*m* = 0.29) was slightly higher than those for saprotrophic (*m* = 0.16) and pathotrophic richness (*m* = 0.17; Figure 3A). The species richness (*q* = 0) of three fungal guilds were influenced by different soil properties, i.e., soil total phosphorus and available phosphorus for the saprotrophic and pathotrophic fungi (Figures 4A,B) and soil pH, clay content and available phosphorus for the symbiotrophic fungi (Figure 4C).

Along the salinity gradient, the community niches of three fungal guilds were not clearly separated (Figure 3B). The community composition of saprotrophic and pathotrophic fungi (e.g., Sorensen index) showed significant Mantel correlation with soil salinity but that of symbiotrophic fungi was not. However, the community composition of three fungal guilds showed no significant partial Mantel correlations with soil salinity (Figure 5; Supplementary Table 11), suggesting the indirect effects of soil salinity on the community composition of saprotrophic and pathotrophic fungi.

### Salinity Regulation on Alpha Diversity of Rare and Abundant Functional Fungi

The species richness (*q* = 0) rather than the diversity of abundant taxa (*q* = 1, 2) for each fungal guild was more susceptible to changes in soil properties (Figure 4). The exponential of Shannon entropy (*q* = 1) and inverse Simpson diversity (*q* = 2) of three fungal guilds showed no significant correlations with soil salinity (Figure 3A). Compared with the species richness (*q* = 0), the inverse Simpson diversities (*q* = 2) of saprotrophic and pathotrophic fungi showed no correlations with soil total phosphorus and available phosphorus (Figures 4A,B; Supplementary Tables 8 and 9). The soil total phosphorus and available phosphorus were positively correlated soil salinity (Supplementary Figure 4). The inverse Simpson diversity (*q* = 2) of symbiotrophic fungi showed no significant correlations with soil pH and clay content (Figure 4C; Supplementary Table 10).

### Effect of Environmental Distances on Composition of Rare and Abundant Functional Fungi

The Sorensen index (*q* = 0) of fungi dissimilarity assigns more weights to rare taxa than the Morisita-Horn index (*q* = 2). The Sorensen index (*q* = 0) of saprotrophic fungi dissimilarity showed higher Mantel and partial Mantel coefficients with soil salinity (*ρ* = 0.098 and *ρ* = 0.043) than those of the Morisita-Horn index (*q* = 2; *ρ* = 0.062 and *ρ* = 0.003). The Sorensen index (*q* = 0) of pathotrophic fungi dissimilarity not Morisita-Horn (*q* = 2) index showed significantly Mantel correlations with soil salinity. Differently, the three indices of symbiotrophic fungi dissimilarity (i.e., Sorensen, Horn, and Morisita-Horn) displayed no Mantel correlation with soil salinity (Figure 5; Supplementary Table 11).

Compared with the Morisita-Horn index (*q* = 2), the Sorensen index (*q* = 0) of saprotrophic fungi dissimilarity was associated with soil available phosphorus (*ρ* = 0.088, *p* < 0.001) and that of pathotrophic fungi dissimilarity was associated with total phosphorus (*ρ* = 0.057, *p* < 0.05). The Sorensen indices (*q* = 0) of saprotrophic and pathotrophic fungi dissimilarity were not associated with soil total nitrogen (*ρ* = 0.034, *p* > 0.05; *ρ* = 0.005, *p* > 0.05; Figures 5A,B; Supplementary Table 11). In contrast, the Sorensen, Horn, and Morisita-Horn indices (*q* = 0, 1, and 2) of symbiotrophic fungi dissimilarity showed similar partial Mantel coefficients with soil total nitrogen (Figure 5C).

## Discussion

### Salinity Promotes the Relative Abundance and Species Richness of Symbiotrophic Fungi Over Saprotrophic Fungi

The relative abundance of symbiotrophic fungi increased along the salinity gradient, but those of saprotrophic and pathotrophic fungi did not change significantly (Figure 2). Furthermore, the species richness of symbiotrophic fungi showed greater increases to soil salinization than the other two fungal guilds. These results partially supported our prediction that the diversity of symbiotrophic fungi increases with soil salinity and that of saprotrophic and pathotrophic fungi decrease along the salinity gradient. Symbiotrophic fungi can establish mutualistic associations with plants and alleviate the negative impacts of salinization on plants by promoting plant K^+^ uptake and antioxidant enzymes activity (Akyol et al., 2020). In this study, 59.9% symbiotrophic fungi were arbuscular mycorrhizal fungi. In high saline conditions, symbiotrophic fungi could assist plants in maintaining their productivity (Bencherif et al., 2015). Although the functions of soil fungi were not measured, the response of functional guilds to soil salinization could provide the implications for ecosystem functions. For instance, the diversity of symbiotrophic fungi greatly prompts plant biodiversity, nutrient uptake, and productivity in microcosm (van der Heijden et al., 1998). So, increasing relative abundance and species richness of symbiotrophic fungi would improve the plant uptake of water and mineral nutrients at high salt concentrations. The significance of symbiotrophic fungi to plant growth increases remarkably at higher salt concentrations (Ren et al., 2016). Similarly, the spores and biomass of symbiotrophic fungi increase with increasing salinity in natural sites (Bencherif et al., 2015). More symbiotrophic fungal taxa are associated with plants and plants are less selective in choosing symbiotrophic fungi under stress compared to the unstressed condition (Lin et al., 2021). Increased symbiotrophic fungi (i.e., relative abundance and species richness) were likely to meet the demand of plants on nutrients and offset the adverse impacts of salinity on plant productivity at high salt concentrations.

In contrast, saprotrophic fungi were the most abundant among three functional fungal guilds and their relative abundance is highly resistant to salinity changes (Figure 2), which has been observed in semi-arid mangroves (Vanegas et al., 2019). It is probably because saprotrophic fungi are actively involved in organic matter decomposition and depend highly on organic inputs. Elmajdoub and Marschner (2015) found that repeated organic matter amendments can alleviate the adverse impacts of salinization on the growth of saprotrophic fungi. In this study, the aboveground plant biomass is similar along the salinity gradient (Supplementary Figure 2), resulting in relatively steady organic matter inputs to soil. Similarly, the saprotrophic fungi to microbial biomass ratio slightly increases with salinity in a plant residue addition experiment (Wichern et al., 2006). The resistance of saprotrophic fungi in terms of relative abundance may be confirmed by the functions, such as litter decomposition and nutrient release in the shrub shelterbelts under soil salinization.

Like saprotrophic fungi, the relative abundance of pathotrophic fungi were not correlated with soil salinity, suggesting that pathogenic fungi either adapt or be resistant to salinity changes. Until now, few studies have investigated the response of pathotrophic fungi to soil salinization and little is known about the reasons. Five species of pathotrophic fungi for sugarbeet seedlings exhibited great tolerance to increasing soil salinity (El-Abyad et al., 1988), agreeing with the result of this study.

The species richness of three fungal guilds all increased with increasing soil salinity but in different magnitudes, and the saprotrophic and pathotrophic fungi showed smaller changes than the symbiotrophic fungi (Figure 3A). We speculate that the increasing species richness of saprotrophic and pathotrophic fungi to salinity changes observed here might be attributed to high plasticity of soil fungi under salinity stress (Rath et al., 2019). The plasticity of soil fungi suggests that fungal individuals, over the course of a lifetime, shift trait expression in dealing with changes in the environment (Coleine et al., 2022). High plasticity of soil fungi may imply great stress tolerance but low growth and death rates (Plemenitas et al., 2014). But we are aware that the plasticity of soil fungi should be measured for active fungal communities by using metagenomics or transcriptomics. Thus, advanced techniques should be used to disentangle the mechanisms that drive different responses of fungal functional guilds to salinity stress in future.

Soil organic carbon (SOC) was an important carbon resource for saprotrophic fungi (Bardgett, 2005) and was significantly positively correlated to soil salinity (Supplementary Figure 4). The increment of SOC implies higher soil porosity and water retention capacity (Pathy et al., 2020), providing shelters for soil fungi. Meanwhile, the impacts of geographic distance and soil depth on fungal community dissimilarity imply the stochastic process and dispersal limitation of soil fungi (Wang et al., 2021). The low dispersal rate of soil fungi could be caused by special fungal propagules and the harsh environmental barriers (Zhou and Ning, 2017). Thus, we reason that in studied sites heterogeneous resource at high-salinity soils due to high SOC content could facilitate the migration and occupation of saprotrophic and pathotrophic fungi. In addition, stressful habitats that limit fungal growth may decrease resource competition and increase fungal diversity by suppressing dominating species (Oono et al., 2020). More taxa of saprotrophic fungi would be coexisted with high soil salinity, because the physiological activity of saprotrophic fungi might be inhibited by increasing salinity (Rath et al., 2019). This demonstrates that salinity stress and SOC resource may jointly shape the species richness of saprotrophic fungi under soil salinization.

### Rare Taxa of Functional Fungi Display Higher Diversity Sensitivity to Soil Salinity

This study showed that the diversity of the rare fungal taxa (*q* = 0) not the abundant taxa (*q* = 1, 2) displayed significant correlations with soil salinity and was more susceptible to other soil properties (Figure 4). The different patterns between the diversities of rare (*q* = 0) and abundant fungal taxa (*q* = 1, 2) indicate that the rare taxa of functional guilds are more sensitive to increasing soil salinity than the abundant taxa. Previous studies found that abundant fungi display great resistance to drought stress and ecosystem disturbance (Sun et al., 2017; de Vries et al., 2018). The activity or growth of abundant fungal taxa might be constricted due to high soil salinity or low-quality carbon resource in the desert soils, which promotes the migration rate and diversity of rare fungal taxa (Rath et al., 2016). The growth of a dominant root fungus (the *Inula viscosa*) was strongly inhibited by soil salinity *in vitro* assays and the fungal diversity increased at sites with high soil salinity (Macia-Vicente et al., 2012). Oono et al. (2020) indicate that abundant fungal taxa were suppressed in stressful conditions, which promoted the population of rare fungal taxa and increased fungal diversity. The population size of rare fungi would be enlarged with increasing SOC along the salinity gradient, because high SOC content provides more niches for rare fungi. Moreover, the great sensitivity of rare taxa of soil functional fungal guilds to salinity changes could attribute to their high variability, as rare taxa could function as a large reservoir of genetic traits and have redundant functions (Boraks et al., 2020; Pascoal et al., 2021). Therefore, the diversity of rare fungal taxa in this study increased with salinity changes.

This study found that the diversity of rare fungal taxa is more sensitive to soil phosphorus compared to that of abundant fungal taxa. Rare fungal taxa that have phosphorus cycling functions in saline soils can be influenced by soil total phosphorus and available phosphorus. The diversity of rare symbiotrophic fungi (*q* = 0) was positively correlated with soil pH as well as soil available phosphorus (Figure 4C). Symbiotrophic fungi are associated with plant P and nitrogen nutrition (Ezawa et al., 2002; Lindahl and Tunlid, 2015). In P-poor soils, such as the desert soil with high pH, the mutualistic association between plant and symbiotrophic fungi may be stimulated, causing a high diversity of rare symbiotrophic fungi. Phosphorus uptake of symbiotrophic fungi was found to increase at higher soil pH (Hinsinger, 2001). Unlike the diversities of abundant taxa (*q* = 1 and 2), the diversity of rare saprotrophic and pathotrophic fungi (*q* = 0) was negatively correlated with soil total phosphorus and available phosphorus (Figures 4A,B), suggesting that rare taxa may play more important roles in phosphorus cycling than abundant species. The diversity of rare saprotrophic and pathotrophic fungi was positively correlated with soil alkaline phosphatase (Supplementary Table 16).

### Community Composition of Functional Fungi Indirectly Influenced by Soil Salinity and Mainly Through Rare Taxa

The community composition of rare saprotrophic and pathotrophic fungi showed higher positive correlations with salinity changes (distance of soil salinity) compared to those of abundant taxa. This result supported the prediction that rare taxa of the functional guilds of soil fungi are more sensitive to salinity changes than the abundant taxa. However, compared with the diversity, the community composition of rare fungal taxa was not significantly correlated with soil salinity partially, suggesting that soil salinity may have indirect effects on community composition of rare fungal taxa. Kim et al. (2019) reported that fungal community composition in the salinity treatment is mainly driven by soil salinity. The impacts of salinity on fungal community composition might be offset by organic matter, as soil salinity and organic carbon were significantly positively correlated (Supplementary Figure 4) and exhibited opposite effects on fungal community composition in a semi-arid mangrovein salt mash (Vanegas et al., 2019).

The community composition of rare saprotrophic and pathotrophic fungi could be indirectly affected by soil salinity through altering soil phosphorus and clay content. Rare taxa of saprotrophic and pathotrophic fungi (i.e., Sorensen index) not the abundant taxa (i.e., Morisita-Horn index) were significantly influenced by changes in soil phosphorus (distance of soil phosphorus; Supplementary Table 11). Similar to this study, the community composition of sapraotrophic and pathotrophic fungi in a natural grassland is mainly driven by soil phosphorus (Delavaux et al., 2021). In current study, soil salinity showed significantly positively correlation with soil phosphorus and clay content and was not correlated with soil total nitrogen, which were controlling factors for the community composition of rare taxa (i.e., Sorensen index). These correlations confirmed that the responses of community composition of saprotrophic and pathotrophic fungi to soil salinization might be induced mainly by rare taxa (i.e., Sorensen index), as abundant taxa have wider variability in abundance under changing resource availability, e.g., soil total nitrogen (Newton and Shade, 2016).

The community composition of rare and abundant symbiotrophic fungi (i.e., Sorensen, Horn, and Morisita-Horn indices) showed no correlations with salinity changes, indicating that salinization had similar stresses on the rare and abundant symbiotrophic fungi. This result partially supported the prediction that the community composition of symbiotrophic fungi is expected to occupy high-salinity niches compared to that of saprotrophic and pathotrophic fungi. The community composition of symbiotrophic fungi was mainly regulated by soil total nitrogen. Symbiotrophic fungi provide mineral nitrogen and phosphorus nutrients to plants in exchange for carbohydrates (Kiers et al., 2011) and enhance nitrogen accumulation in plants with inreasing soil salinity (Zhu et al., 2018), as the accumulation of leaf non-protein nitrogen increase plant resistance to salinity (Mansour, 2000). Given that the leaf N content and N:P ratio of *H. ammodendron* at high-salinity sites were significantly higher than those at low-salinity sites (Feng et al., in preparation; Supplementary Figure 5), symbiotrophic fungi that provide nitrogen would be promoted with increasing salinity due to plant selection, especially at the high-salinity sites. Furthermore, different symbiotrophic fungi can acquire N from distinctly sources, e.g., ammonium N and nitrate N (Cox et al., 2010) that are influenced by soil total N (Supplementary Figure 6). Therefore, the community composition of symbiotrophic fungi was driven by soil total N under salt stress and resistant to changes in soil salinity.

### Conclusion

Soil fungal communities are integral to soil carbon cycling, plant nutrition, and pathogenicity. This study indicates that the rare taxa of soil functional fungal guilds (i.e., saprotrophic, pathotrophic, and symbiotrophic fungi) rather than the abundant taxa would be more sensitive to changes in soil salinity, thus the biodiversity responses of fungal guilds to soil salinization should be reflected by rare taxa. Changes in soil salinity increased the diversity of rare saprotrophic and pathotrophic fungal taxa and indirectly affected their community composition. Compared to saprotrophic and pathotrophic fungi, symbiotrophic fungi increased more in alpha diversity but did not significantly change the community composition with increasing soil salinity. These results advance our understanding of the responses of rare taxa of functional fungal guilds to soil salinization and contribute to soil biodiversity conservation and soil health management regarding fugal functionality in the inland saline environments.

## Data Availability Statement

The datasets presented in this study can be found in online repositories. The names of the repository/repositories and accession number(s) can be found at: European Nucleotide Archive (ENA) - PRJEB46878.

## Author Contributions

LL: methodology, investigation, formal analysis, writing-original draft, and writing-review and editing. XJ: methodology, investigation, formal analysis, review, and editing. ML-B and CS: review and editing. YW: investigation. WF: methodology, investigation, and writing-review and editing. All authors contributed to the article and approved the submitted version.

## Funding

The financial support for this work is the Agricultural Science and Technology Innovation Program of Chinese Academy of Agricultural Sciences, the National Natural Science Foundation of China (41730638 and U1803342), and K.C. Wong Education Foundation.

## Conflict of Interest

The authors declare that the research was conducted in the absence of any commercial or financial relationships that could be construed as a potential conflict of interest.

## Publisher’s Note

All claims expressed in this article are solely those of the authors and do not necessarily represent those of their affiliated organizations, or those of the publisher, the editors and the reviewers. Any product that may be evaluated in this article, or claim that may be made by its manufacturer, is not guaranteed or endorsed by the publisher.

## Footnotes

^1^ http://www.usadellab.org/cms/?page=trimmomatic

^2^ https://ccb.jhu.edu/software/FLASH/

^3^ http://www.ebi.ac.uk/ena/data/view/OU495976-OU501470
