# Supplementary material for: Rare Taxa Drive the Response of Soil Fungal Guilds to Soil Salinization in the Taklamakan Desert
Source: Front Microbiol. 2022 May 23;13:862245. doi: 10.3389/fmicb.2022.862245 (PMC9168468; doi:10.3389/fmicb.2022.862245)
Supplement: Supplementary file 1 [file Data_Sheet_1.doc]

**Supplemental materials**

Supplemental tables: 16 tables

Supplemental figures: 6 figures

**Supplemental Table 1. Relative abundance of functional guilds of soil fungi. Mean (standard error), N=18. Different letters in columns denote statistically significant differences among sites. The sites' ID in the first column denote sampling sites from the north to the middle of Tarim desert highway.**

| Sites | Saprotrophic fungi | Pathotrophic fungi | Symbiotrophic fungi | Unassigned fungi |
| --- | --- | --- | --- | --- |
| S01 | 29.0(4.9) ab | 8.0(1.77) bc | 0.2(0.0) b | 62.8(5.9) ab |
| S04 | 25.0(4.1) ab | 9.7(2.37) bc | 0.3(0.1) b | 65.0(5.2) ab |
| S07 | 15.0(3.6) b | 4.3(1.3) c | 0.5(0.1) b | 80.2(4.2) a |
| S10 | 30.3(5.9) ab | 9.4(2.2) bc | 1.7 (0.7) a | 58.6(5.8) ab |
| S13 | 12.8 (2.1) b | 7.3(2.9) bc | 0.4(0.2) b | 79.6(3.3) a |
| S16 | 29.1(5.7) ab | 6.2(1.3) bc | 0.2(0.0) b | 64.6(6.5) ab |
| S19 | 18.1(5.2) b | 28.1(6.2) a | 0.2(0.0) b | 53.7(7.2) ab |
| S23 | 42.6(6.5) ab | 7.7(2.1) bc | 0.2(0.1) b | 49.6(6.8) b |
| S28 | 18.9(3.8) b | 19.6(4.3) ab | 0.4(0.2) b | 61.2(6.8) ab |
| S34 | 23.7(3.7) ab | 23.2(3.5) ab | 0.7(0.3) ab | 52.4(5.0) ab |
| All | 25.1 | 12.0 | 0.5 | 62.4 |

**Supplemental Table 2.** Three methods for estimating diversity response of rare and abundant taxa to changing environment.

| Method | Description | Unique information | Limitation | Reference |
| --- | --- | --- | --- | --- |
| Relative abundance | Abundance above or below 0.01%, 0.1%, 0.31% of all samples | Classify abundant and rare taxa based on thresholds | Depend on sequencing depth and number of sampling sies | (L*iu et a*l., 2015; Lynch & Neufeld, 2015; Oo*no et a*l., 2017) |
| Frequency | Estimate probability (frequency) to be abundant or rare | Seeks to place species along a rarity-commonness gradient | Not applied to communities composed of ≤5 species; sampling scale | (Balbue*na et a*l., 2021) |
| Alpha-diversity  metrices | Weights assignment to rare and abundant taxa | Effective number of rare or abundant species | Not clearly classify abundant and rare taxa | (Gossner et al., 2016; *Xu et a*l., 2020) |

**Supplemental Table 3. The effects of soil properties and interactions between soil salinity and depth on the relative abundance of soil saprotrophic, pathotrophic, and symbiotrophic fungi. The sampling site was considered as a random factor. Values in bold are significant at alpha level = 0.05. Salinity = soil salinity, Depth = soil depth, STN = soil total nitrogen, Clay = soil clay content, STP = soil total phosphorus, avaP = soil available phosphorus.**

| Saprotrophic fungi | | | |  |
| --- | --- | --- | --- | --- |
| Marginal *R*^2^= 0.062 | | Conditional *R*^2^= 0.191 | |  |
|  | *F* | *Pr*(>*F*) | Chisq | *Pr*(>Chisq) |
| Salinity | 0.502 | 0.480 | 0.000 | 0.984 |
| Depth | 0.695 | 0.628 | 5.716 | 0.335 |
| pH | 0.964 | 0.329 | 0.964 | 0.326 |
| Clay | 0.690 | 0.407 | 0.690 | 0.406 |
| STN | 0.004 | 0.951 | 0.004 | 0.951 |
| STP | 0.007 | 0.932 | 0.007 | 0.932 |
| avaP | 0.239 | 0.625 | 0.239 | 0.625 |
| Salinity:Depth | 0.392 | 0.853 | 1.962 | 0.854 |
| Pathotrophic fungi | | | |  |
| Marginal *R*^2^= 0.072 | | Conditional *R*^2^= 0.365 | |  |
|  | *F* | *Pr*(>*F*) | Chisq | *Pr*(>Chisq) |
| Salinity | 0.502 | 0.480 | 0.000 | 0.984 |
| Depth | 0.695 | 0.628 | 5.716 | 0.335 |
| pH | 0.964 | 0.329 | 0.964 | 0.326 |
| Clay | 0.690 | 0.407 | 0.690 | 0.406 |
| STN | 0.004 | 0.951 | 0.004 | 0.951 |
| STP | 0.007 | 0.932 | 0.007 | 0.932 |
| avaP | 0.239 | 0.625 | 0.239 | 0.625 |
| Salinity:Depth | 0.392 | 0.853 | 1.962 | 0.854 |
| Symbiotrophic fungi | | | |  |
| Marginal *R*^2^= 0.067 | | Conditional *R*^2^= 0.317 | |  |
|  | *F* | *Pr*(>*F*) | Chisq | *Pr*(>Chisq) |
| Salinity | 1.047 | 0.308 | **4.008** | **0.045** |
| Depth | 0.957 | 0.446 | 2.752 | 0.738 |
| pH | 1.694 | 0.195 | 1.694 | 0.193 |
| Clay | 0.164 | 0.686 | 0.164 | 0.686 |
| STN | 0.289 | 0.592 | 0.289 | 0.591 |
| STP | 0.000 | 0.998 | 0.000 | 0.998 |
| avaP | 0.801 | 0.372 | 0.801 | 0.371 |
| Salinity:Depth | 0.729 | 0.603 | 3.644 | 0.602 |

**Supplemental Table 4. The effects of soil properties and interactions between soil salinity and depth on the alpha diversity of saprotrophic fungi. The sampling site was considered as a random factor. Values in bold are significant at alpha level = 0.05. Salinity = soil salinity, Depth = soil depth, Clay = soil clay content, STN = soil total nitrogen, STP = soil total phosphorus, avaP = soil available phosphorus.**

| Species richness (*q*= 0) | | | |  |
| --- | --- | --- | --- | --- |
| Marginal *R*^2^= 0.112 | | Conditional *R*^2^= 0.241 | |  |
|  | *F* | *Pr*(>*F*) | Chisq | *Pr*(>Chisq) |
| Salinity | 3.015 | 0.087 | **11.001** | **0.001** |
| Depth | 0.617 | 0.687 | 3.468 | 0.628 |
| pH | 0.810 | 0.370 | 0.810 | 0.368 |
| Clay | 0.341 | 0.560 | 0.341 | 0.559 |
| STN | 0.140 | 0.709 | 0.140 | 0.709 |
| STP | **5.997** | **0.016** | **5.997** | **0.014** |
| avaP | 2.900 | 0.090 | 2.900 | 0.089 |
| Salinity:Depth | 0.264 | 0.932 | 1.319 | 0.933 |
| Exponential of Shannon entropy (*q*= 1) | | | |  |
| Marginal *R*^2^= 0.151 | | Conditional *R*^2^= 0.328 | |  |
|  | *F* | *Pr*(>*F*) | Chisq | *Pr*(>Chisq) |
| Salinity | 5.055 | 0.028 | 3.099 | 0.078 |
| Depth | 0.736 | 0.598 | 2.097 | 0.836 |
| pH | 0.060 | 0.807 | 0.060 | 0.806 |
| Clay | 1.440 | 0.232 | 1.440 | 0.230 |
| STN | **4.221** | **0.042** | **4.221** | **0.040** |
| STP | 2.166 | 0.143 | 2.166 | 0.141 |
| avaP | 1.017 | 0.315 | 1.017 | 0.313 |
| Salinity:Depth | 1.098 | 0.364 | 5.492 | 0.359 |
| Inverse Simpson (*q*= 2) | | | |  |
| Marginal *R*^2^= 0.142 | | Conditional *R*^2^= 0.277 | |  |
|  | *F* | *Pr*(>*F*) | Chisq | *Pr*(>Chisq) |
| Salinity | 5.899 | 0.019 | 3.396 | 0.065 |
| Depth | 0.614 | 0.689 | 1.859 | 0.868 |
| pH | 0.028 | 0.868 | 0.028 | 0.868 |
| Clay | 1.607 | 0.207 | 1.607 | 0.205 |
| STN | **4.481** | **0.036** | **4.481** | **0.034** |
| STP | 1.620 | 0.206 | 1.620 | 0.203 |
| avaP | 0.274 | 0.601 | 0.274 | 0.601 |
| Salinity:Depth | 1.325 | 0.257 | 6.626 | 0.250 |

**Supplemental Table 5. The effects of soil properties and interactions between soil salinity and depth on the alpha diversity of pathotrophic fungi. The sampling site was considered as a random factor. Values in bold are significant at alpha level = 0.05. Salinity = soil salinity, Depth = soil depth, Clay = soil clay content, STN = soil total nitrogen, STP = soil total phosphorus, avaP = soil available phosphorus.**

| Species richness (*q*= 0) | | | |  |
| --- | --- | --- | --- | --- |
| Marginal *R*^2^= 0.157 | | Conditional *R*^2^= 0.313 | |  |
|  | *F* | *Pr*(>*F*) | Chisq | *Pr*(>Chisq) |
| Salinity | 0.693 | 0.408 | **10.707** | **0.001** |
| Depth | 0.865 | 0.506 | 4.319 | 0.505 |
| pH | 0.215 | 0.644 | 0.215 | 0.643 |
| Clay | 0.993 | 0.321 | 0.993 | 0.319 |
| STN | 0.446 | 0.505 | 0.446 | 0.504 |
| STP | 3.350 | 0.069 | 3.350 | 0.067 |
| avaP | **4.356** | **0.038** | **4.356** | **0.037** |
| Salinity:Depth | 1.036 | 0.399 | 5.180 | 0.394 |
| Exponential of Shannon entropy (*q*= 1) | | | |  |
| Marginal *R*^2^= 0.122 | | Conditional *R*^2^= 0.173 | |  |
|  | *F* | *Pr*(>*F*) | Chisq | *Pr*(>Chisq) |
| Salinity | 3.846 | 0.058 | 0.707 | 0.400 |
| Depth | 2.060 | 0.074 | 8.344 | 0.138 |
| pH | 0.000 | 0.982 | 0.000 | 0.982 |
| Clay | 0.593 | 0.443 | 0.593 | 0.441 |
| STN | 1.242 | 0.268 | 1.242 | 0.265 |
| STP | 0.050 | 0.824 | 0.050 | 0.823 |
| avaP | 0.359 | 0.550 | 0.359 | 0.549 |
| Salinity:Depth | 0.978 | 0.433 | 4.890 | 0.429 |
| Inverse Simpson (*q*= 2) | | | |  |
| Marginal *R*^2^= 0.116 | | Conditional *R*^2^= 0.164 | |  |
|  | *F* | *Pr*(>*F*) | Chisq | *Pr*(>Chisq) |
| Salinity | 3.491 | 0.070 | 0.220 | 0.639 |
| Depth | 1.846 | 0.107 | 7.015 | 0.220 |
| pH | 0.004 | 0.951 | 0.004 | 0.951 |
| Clay | 0.785 | 0.378 | 0.785 | 0.376 |
| STN | 0.844 | 0.361 | 0.844 | 0.358 |
| STP | 0.026 | 0.873 | 0.026 | 0.873 |
| avaP | 0.458 | 0.500 | 0.458 | 0.499 |
| Salinity:Depth | 1.025 | 0.405 | 5.127 | 0.401 |

**Supplemental Table 6. The effects of soil properties and interactions between soil salinity and depth on the alpha diversity of symbiotrophic fungi. The sampling site was considered as a random factor. Values in bold are significant at alpha level = 0.05. Salinity = soil salinity, Depth = soil depth, Clay = soil clay content, STN = soil total nitrogen, STP = soil total phosphorus, avaP = soil available phosphorus.**

| Species richness (*q*= 0) | | | |  |
| --- | --- | --- | --- | --- |
| Marginal *R*^2^= 0.126 | | Conditional *R*^2^= 0.309 | |  |
|  | *F* | *Pr*(>*F*) | Chisq | *Pr*(>Chisq) |
| Salinity | **0.407** | **0.525** | **4.723** | **0.030** |
| Depth | 0.925 | 0.466 | 5.192 | 0.393 |
| pH | **4.451** | **0.037** | **4.451** | **0.035** |
| Clay | 3.009 | 0.085 | 3.009 | 0.083 |
| STN | 0.293 | 0.589 | 0.293 | 0.589 |
| STP | 2.601 | 0.109 | 2.601 | 0.107 |
| avaP | **9.444** | **0.002** | **9.444** | **0.002** |
| Salinity:Depth | 0.287 | 0.919 | 1.437 | 0.920 |
| Exponential of Shannon entropy (*q*= 1) | | | |  |
| Marginal *R*^2^= 0.098 | | Conditional *R*^2^= 0.262 | |  |
|  | *F* | *Pr*(>*F*) | Chisq | *Pr*(>Chisq) |
| Salinity | 0.001 | 0.980 | 0.593 | 0.441 |
| Depth | 0.672 | 0.645 | 3.882 | 0.567 |
| pH | 3.362 | 0.069 | 3.362 | 0.067 |
| Clay | 2.710 | 0.102 | 2.710 | 0.100 |
| STN | 0.180 | 0.672 | 0.180 | 0.672 |
| STP | 1.272 | 0.261 | 1.272 | 0.259 |
| avaP | **7.581** | **0.007** | **7.581** | **0.006** |
| Salinity:Depth | 0.554 | 0.735 | 2.770 | 0.735 |
| Inverse Simpson (*q*= 2) | | | |  |
| Marginal *R*^2^= 0.074 | | Conditional *R*^2^= 0.169 | |  |
|  | *F* | *Pr*(>*F*) | Chisq | *Pr*(>Chisq) |
| Salinity | 0.258 | 0.613 | 0.073 | 0.788 |
| Depth | 0.510 | 0.769 | 2.284 | 0.809 |
| pH | 1.287 | 0.259 | 1.287 | 0.257 |
| Clay | 2.788 | 0.097 | 2.788 | 0.095 |
| STN | 0.216 | 0.643 | 0.216 | 0.642 |
| STP | 0.364 | 0.547 | 0.364 | 0.546 |
| avaP | **5.377** | **0.022** | **5.377** | **0.020** |
| Salinity:Depth | 0.925 | 0.467 | 4.625 | 0.463 |

**Supplemental Table 7. The effects of soil properties on the relative abundance of soil saprotrophic, pathotrophic, and symbiotrophic fungi. Soil depth and sampling site were considered as random factors. Values in bold are significant at alpha level = 0.05. Salinity = soil salinity, Depth = soil depth, STN = soil total nitrogen, Clay = soil clay content, STP = soil total phosphorus, avaP = soil available phosphorus.**

| Saprotrophic fungi | | | | | |
| --- | --- | --- | --- | --- | --- |
| Marginal *R*^2^= 0.024 | | Conditional *R*^2^= 0.178 | |  |  |
|  | Estimate | *t* | *Pr*(>|*t*|) | Chisq | *Pr*(>Chisq) |
| Salinity | 0.006 | 0.070 | 0.944 | 0.005 | 0.944 |
| pH | 0.019 | 0.215 | 0.830 | 0.046 | 0.830 |
| Clay | 0.088 | 1.043 | 0.298 | 1.089 | 0.297 |
| STN | 0.015 | 0.162 | 0.872 | 0.026 | 0.872 |
| STP | 0.013 | 0.138 | 0.891 | 0.019 | 0.890 |
| avaP | 0.099 | 1.187 | 0.237 | 1.409 | 0.235 |
|  |  |  |  |  |  |
| Pathotrophic fungi | | | | | |
| Marginal *R*^2^= 0.051 | | Conditional *R*^2^= 0.358 | |  |  |
|  | Estimate | *t* | *Pr*(>|*t*|) | Chisq | *Pr*(>Chisq) |
| Salinity | 0.128 | 1.584 | 0.115 | 2.510 | 0.113 |
| pH | -0.072 | -0.814 | 0.417 | 0.663 | 0.416 |
| Clay | 0.017 | 0.209 | 0.835 | 0.044 | 0.835 |
| STN | -0.014 | -0.163 | 0.871 | 0.027 | 0.871 |
| STP | -0.212 | **-2.219** | **0.028** | **4.923** | **0.027** |
| avaP | -0.008 | -0.104 | 0.918 | 0.011 | 0.917 |
|  |  |  |  |  |  |
| Symbiotrophic fungi | | | | | |
| Marginal *R*^2^= 0.049 | | Conditional *R*^2^= 0.285 | |  |  |
|  | Estimate | *t* | *Pr*(>|*t*|) | Chisq | *Pr*(>Chisq) |
| Salinity | 0.173 | **2.100** | **0.037** | **4.412** | **0.036** |
| pH | -0.147 | -1.648 | 0.101 | 2.716 | 0.099 |
| Clay | -0.021 | -0.256 | 0.798 | 0.066 | 0.798 |
| STN | 0.036 | 0.401 | 0.689 | 0.161 | 0.688 |
| STP | -0.039 | -0.405 | 0.686 | 0.164 | 0.686 |
| avaP | -0.057 | -0.704 | 0.483 | 0.495 | 0.482 |

**Supplemental Table 8. The effects of soil properties on the alpha diversity of saprotrophic fungi. Soil depth and sampling site were considered as random factors. Values in bold are significant at alpha level = 0.05. Salinity = soil salinity, Clay = soil clay content, STN = soil total nitrogen, STP = soil total phosphorus, avaP = soil available phosphorus.**

| Species richness (*q*= 0) | | | | | |
| --- | --- | --- | --- | --- | --- |
| Marginal *R*^2^= 0.096 | | Conditional *R*^2^= 0.223 | |  |  |
|  | Estimate | *t* | *Pr*(>|*t*|) | Chisq | *Pr*(>Chisq) |
| Salinity | 0.161 | **3.120** | **0.002** | **9.739** | **0.002** |
| pH | 0.039 | 0.880 | 0.383 | 0.767 | 0.381 |
| Clay | 0.016 | 0.370 | 0.714 | 0.135 | 0.714 |
| STN | -0.011 | -0.240 | 0.809 | 0.058 | 0.809 |
| STP | -0.151 | **-2.920** | **0.004** | **8.536** | **0.003** |
| avaP | -0.087 | **-2.060** | **0.040** | **4.264** | **0.039** |
|  |  |  |  |  |  |
| Exponential of Shannon entropy (*q*= 1) | | | | | |
| Marginal *R*^2^= 0.123 | | Conditional *R*^2^= 0.326 | |  |  |
|  | Estimate | *t* | *Pr*(>|*t*|) | Chisq | *Pr*(>Chisq) |
| Salinity | 0.145 | 1.530 | 0.128 | 2.336 | 0.126 |
| pH | 0.011 | 0.140 | 0.893 | 0.018 | 0.893 |
| Clay | -0.132 | -1.670 | 0.097 | 2.782 | 0.095 |
| STN | -0.193 | **-2.250** | **0.026** | **5.074** | **0.024** |
| STP | -0.144 | -1.510 | 0.132 | 2.292 | 0.130 |
| avaP | -0.119 | -1.560 | 0.122 | 2.421 | 0.120 |
|  |  |  |  |  |  |
| Inverse Simpson (*q*= 2) | | | | | |
| Marginal *R*^2^= 0.111 | | Conditional *R*^2^= 0.271 | |  |  |
|  | Estimate | *t* | *Pr*(>|*t*|) | Chisq | *Pr*(>Chisq) |
| Salinity | 0.145 | 1.530 | 0.128 | 2.336 | 0.126 |
| pH | 0.011 | 0.140 | 0.893 | 0.018 | 0.893 |
| Clay | -0.132 | -1.670 | 0.097 | 2.782 | 0.095 |
| STN | -0.193 | **-2.250** | **0.026** | **5.074** | **0.024** |
| STP | -0.144 | -1.510 | 0.132 | 2.292 | 0.130 |
| avaP | -0.119 | -1.560 | 0.122 | 2.421 | 0.120 |

**Supplemental Table 9.** The effects of soil properties on the alpha diversity of pathotrophic fungi. Soil depth and sampling site were considered as random factors. Values in bold are significant at alpha level = 0.05. Salinity = soil salinity, Clay = soil clay content, STN = soil total nitrogen, STP = soil total phosphorus, avaP = soil available phosphorus.

| Species richness (*q*= 0) | | | | | |
| --- | --- | --- | --- | --- | --- |
| Marginal *R*^2^= 0.131 | | Conditional *R*^2^= 0.277 | |  |  |
|  | Estimate | *t* | *Pr*(>|*t*|) | Chisq | *Pr*(>Chisq) |
| Salinity | 0.169 | **2.670** | **0.008** | **7.141** | **0.008** |
| pH | 0.078 | 1.420 | 0.156 | 2.031 | 0.154 |
| Clay | -0.078 | -1.470 | 0.144 | 2.159 | 0.142 |
| STN | -0.035 | -0.610 | 0.542 | 0.374 | 0.541 |
| STP | -0.177 | **-2.780** | **0.006** | **7.756** | **0.005** |
| avaP | -0.135 | **-2.630** | **0.009** | **6.900** | **0.009** |
|  |  |  |  |  |  |
| Exponential of Shannon entropy (*q*= 1) | | | | | |
| Marginal *R*^2^= 0.067 | | Conditional *R*^2^= 0.184 | |  |  |
|  | Estimate | *t* | *Pr*(>|*t*|) | Chisq | *Pr*(>Chisq) |
| Salinity | -0.009 | -0.090 | 0.932 | 0.016 | 0.900 |
| pH | 0.087 | 0.900 | 0.373 | 0.960 | 0.327 |
| Clay | -0.164 | -1.770 | 0.079 | 3.211 | 0.073 |
| STN | -0.070 | -0.710 | 0.482 | 0.480 | 0.488 |
| STP | 0.048 | 0.430 | 0.665 | 0.199 | 0.656 |
| avaP | -0.131 | -1.450 | 0.149 | 2.201 | 0.138 |
|  |  |  |  |  |  |
| Inverse Simpson (*q*= 2) | | | | | |
| Marginal *R*^2^= 0.066 | | Conditional *R*^2^= 0.163 | |  |  |
|  | Estimate | *t* | *Pr*(>|*t*|) | Chisq | *Pr*(>Chisq) |
| Salinity | -0.043 | -0.430 | 0.670 | 0.182 | 0.669 |
| pH | 0.043 | 0.490 | 0.620 | 0.243 | 0.622 |
| Clay | -0.156 | -1.820 | 0.070 | 3.330 | 0.068 |
| STN | -0.041 | -0.450 | 0.660 | 0.200 | 0.655 |
| STP | 0.083 | 0.830 | 0.410 | 0.687 | 0.407 |
| avaP | -0.130 | -1.560 | 0.120 | 2.435 | 0.119 |

**Supplemental Table 10. The effects of soil properties on the alpha diversity of symbiotrophic fungi. Soil depth and sampling site were considered as random factors. Values in bold are significant at alpha level = 0.05. Salinity = soil salinity, Clay = soil clay content, STN = soil total nitrogen, STP = soil total phosphorus, avaP = soil available phosphorus.**

| Species richness (*q*= 0) | | | | | |
| --- | --- | --- | --- | --- | --- |
| Marginal *R*^2^= 0.109 | | Conditional *R*^2^= 0.288 | |  |  |
|  | Estimate | *t* | *Pr*(>|*t*|) | Chisq | *Pr*(>Chisq) |
| Salinity | 0.285 | **2.920** | **0.004** | **8.504** | **0.004** |
| pH | 0.209 | **2.460** | **0.015** | **6.043** | **0.014** |
| Clay | 0.180 | **2.170** | **0.031** | **4.725** | **0.030** |
| STN | 0.093 | 1.050 | 0.297 | 1.097 | 0.295 |
| STP | -0.164 | -1.660 | 0.099 | 2.762 | 0.097 |
| avaP | -0.211 | **-2.670** | **0.008** | **7.148** | **0.008** |
|  |  |  |  |  |  |
| Exponential of Shannon entropy (*q*= 1) | | | | | |
| Marginal *R*^2^= 0.069 | | Conditional *R*^2^= 0.215 | |  |  |
|  | Estimate | *t* | *Pr*(>|*t*|) | Chisq | *Pr*(>Chisq) |
| Salinity | 0.106 | 1.280 | 0.203 | 1.632 | 0.201 |
| pH | 0.150 | **2.060** | **0.041** | **4.258** | **0.039** |
| Clay | 0.144 | **2.020** | **0.045** | **4.093** | **0.043** |
| STN | 0.056 | 0.740 | 0.462 | 0.543 | 0.461 |
| STP | -0.095 | -1.140 | 0.258 | 1.289 | 0.256 |
| avaP | -0.159 | **-2.350** | **0.020** | **5.515** | **0.019** |
|  |  |  |  |  |  |
| Inverse Simpson (*q*= 2) | | | | | |
| Marginal *R*^2^= 0.049 | | Conditional *R*^2^= 0.180 | |  |  |
|  | Estimate | *t* | *Pr*(>|*t*|) | Chisq | *Pr*(>Chisq) |
| Salinity | 0.040 | 0.500 | 0.618 | 0.250 | 0.617 |
| pH | 0.098 | 1.420 | 0.158 | 2.013 | 0.156 |
| Clay | 0.131 | 1.940 | 0.054 | 3.771 | 0.052 |
| STN | 0.041 | 0.560 | 0.575 | 0.317 | 0.574 |
| STP | -0.062 | -0.780 | 0.438 | 0.606 | 0.436 |
| avaP | -0.130 | **-2.000** | **0.047** | **4.008** | **0.045** |

**Supplemental Table 11. Simple and partial Mantel tests for the correlations of changes in the composition of functional guilds of soil fungi with changes in geographic distance and differences in soil properties. Values in the table are the Spearman correction coefficients (rho). Values in bold are significant at alpha level = 0.05. ***: *P* < 0.001; **: *P* < 0.01; *: *P* < 0.05. GD = geographic distance, Salinity = soil salinity, Clay = soil clay content, STN = soil total nitrogen, STP = soil total phosphorus, avaP = soil available phosphorus, Depth = soil depth.**

| Community composition |  | Saprotrophic fungi | | Pathotrophic fungi | | Symbiotrophic fungi | |
| --- | --- | --- | --- | --- | --- | --- | --- |
|  |  | *Simple* | *Partial* | *Simple* | *Partial* | *Simple* | *Partial* |
| Sorensen index (*q*= 0) | GD | **0.231^***^** | **0.212^***^** | **0.242^***^** | **0.229^***^** | **0.247^***^** | **0.235^***^** |
|  | Salinity | **0.098^**^** | 0.043 | **0.090^**^** | 0.042 | -0.021 | -0.051 |
|  | pH | 0.030 | -0.010 | 0.003 | -0.026 | 0.009 | -0.002 |
|  | Clay | **0.105^***^** | **0.080^***^** | **0.072^***^** | **0.044^*^** | 0.036 | 0.025 |
|  | STN | **0.077^*^** | 0.034 | **0.049^*^** | 0.005 | **0.154^***^** | **0.124^**^** |
|  | STP | **0.060^*^** | 0.021 | **0.090^***^** | **0.057^*^** | 0.034 | -0.001 |
|  | avaP | **0.112^**^** | **0.088^**^** | 0.044 | 0.023 | -0.052 | -0.064 |
|  | Depth | **0.042^*^** | 0.015 | **0.067^***^** | **0.051^**^** | 0.035 | **0.050^*^** |
| Horn index (*q*= 1) | GD | **0.217^***^** | **0.204^***^** | **0.304^***^** | **0.298^***^** | **0.275^***^** | **0.265^***^** |
|  | Salinity | **0.071^*^** | 0.007 | 0.034 | -0.009 | -0.033 | -0.064 |
|  | pH | **0.045^*^** | 0.011 | -0.022 | -0.049 | 0.004 | -0.007 |
|  | Clay | **0.190^***^** | **0.170^***^** | **0.076^**^** | **0.058^*^** | 0.034 | 0.026 |
|  | STN | **0.096^**^** | **0.060^*^** | **0.099^**^** | **0.063^*^** | **0.158^***^** | **0.128^***^** |
|  | STP | 0.014 | -0.035 | 0.026 | -0.018 | 0.021 | -0.015 |
|  | avaP | **0.060^*^** | 0.024 | -0.025 | -0.039 | -0.068 | -0.077 |
|  | Depth | **0.089^***^** | **0.066^***^** | **0.057^**^** | **0.066^**^** | 0.033 | **0.055^*^** |
| Morisita–Horn index (*q*= 2) | GD | **0.194^***^** | **0.180^***^** | **0.292^***^** | **0.285^***^** | **0.262^***^** | **0.253^***^** |
|  | Salinity | **0.062^*^** | 0.003 | 0.027 | -0.014 | -0.035 | -0.064 |
|  | pH | 0.044 | 0.015 | -0.018 | -0.042 | 0.001 | -0.009 |
|  | Clay | **0.186^***^** | **0.166^***^** | **0.071^**^** | **0.053^*^** | 0.029 | 0.021 |
|  | STN | **0.097^**^** | **0.064^*^** | **0.104^**^** | **0.069^*^** | **0.153^***^** | **0.124^**^** |
|  | STP | 0.018 | -0.028 | 0.034 | -0.007 | 0.018 | -0.017 |
|  | avaP | 0.046 | 0.010 | -0.031 | -0.045 | -0.064 | -0.071 |
|  | Depth | **0.086^***^** | **0.064^**^** | **0.058^**^** | **0.066^***^** | 0.030 | **0.052^*^** |

**Supplemental Table 12. The effects of soil properties on the species richness of rare and abundant saprotrophic fungi. Rare fungi, relative abundance below 0.01%; Abundant fungi, relative abundance above 0.01%. Soil depth and sampling site were considered as random factors. Values in bold are significant at alpha level = 0.05. Salinity = soil salinity, Clay = soil clay content, STN = soil total nitrogen, STP = soil total phosphorus, avaP = soil available phosphorus.**

| Abundant fungi | | | | | |
| --- | --- | --- | --- | --- | --- |
| Marginal *R*^2^= 0.136 | | Conditional *R*^2^= 0.356 | |  |  |
|  | Estimate | *t* | *Pr*(>|*t*|) | Chisq | *Pr*(>Chisq) |
| Salinity | **0.111** | **3.747** | **0.000** | **14.038** | **0.000** |
| pH | 0.018 | 0.605 | 0.546 | 0.365 | 0.545 |
| Clay | 0.019 | 0.800 | 0.425 | 0.641 | 0.423 |
| STN | -0.021 | -0.815 | 0.416 | 0.664 | 0.415 |
| STP | **-0.073** | **-2.580** | **0.011** | **6.659** | **0.010** |
| avaP | **-0.062** | **-2.721** | **0.007** | **7.404** | **0.007** |
|  |  |  |  |  |  |
| Rare fungi | | | | | |
| Marginal *R*^2^= 0.062 | | Conditional *R*^2^= 0.203 | |  |  |
|  | Estimate | *t* | *Pr*(>|*t*|) | Chisq | *Pr*(>Chisq) |
| Salinity | **0.152** | **2.145** | **0.034** | **4.601** | **0.032** |
| pH | 0.097 | 1.385 | 0.169 | 1.918 | 0.166 |
| Clay | 0.017 | 0.299 | 0.765 | 0.090 | 0.765 |
| STN | 0.014 | 0.216 | 0.830 | 0.047 | 0.829 |
| STP | **-0.162** | **-2.379** | **0.019** | **5.661** | **0.017** |
| avaP | -0.035 | -0.611 | 0.542 | 0.373 | 0.541 |

**Supplemental Table 13. The effects of soil properties on the species richness of rare and abundant pathotrophic fungi. Rare fungi, relative abundance below 0.01%; Abundant fungi, relative abundance above 0.01%. Soil depth and sampling site were considered as random factors. Values in bold are significant at alpha level = 0.05. Relative abundance, the 0.01% threshold approach; frequency, the frequency approach. Salinity = soil salinity, Clay = soil clay content, STN = soil total nitrogen, STP = soil total phosphorus, avaP = soil available phosphorus.**

| Abundant fungi | | | | | |
| --- | --- | --- | --- | --- | --- |
| Marginal *R*^2^= 0.161 | | Conditional *R*^2^= 0.370 | |  |  |
|  | Estimate | *t* | *Pr*(>|*t*|) | Chisq | *Pr*(>Chisq) |
| Salinity | **0.108** | **3.577** | **0.000** | **12.793** | **0.000** |
| pH | 0.029 | 0.943 | 0.348 | 0.888 | 0.346 |
| Clay | -0.013 | -0.525 | 0.601 | 0.275 | 0.600 |
| STN | -0.019 | -0.700 | 0.485 | 0.490 | 0.484 |
| STP | **-0.080** | **-2.761** | **0.006** | **7.622** | **0.006** |
| avaP | **0.108** | **3.577** | **0.000** | **12.793** | **0.000** |
|  |  |  |  |  |  |
| Rare fungi | | | | | |
| Marginal *R*^2^= 0.062 | | Conditional *R*^2^= 0.203 | |  |  |
|  | Estimate | *t* | *Pr*(>|*t*|) | Chisq | *Pr*(>Chisq) |
| Salinity | **0.199** | **2.737** | **0.007** | **7.493** | **0.006** |
| pH | 0.023 | 0.319 | 0.750 | 0.102 | 0.749 |
| Clay | -0.045 | -0.749 | 0.455 | 0.561 | 0.454 |
| STN | -0.050 | -0.773 | 0.441 | 0.597 | 0.440 |
| STP | -0.128 | -1.834 | 0.069 | 3.365 | 0.067 |
| avaP | -0.061 | -1.048 | 0.296 | 1.099 | 0.295 |

**Supplemental Table 14. The effects of soil properties on the species richness of rare and abundant symbiotrophic fungi. Rare fungi, relative abundance below 0.01%; Abundant fungi, relative abundance above 0.01%. Soil depth and sampling site were considered as random factors. Values in bold are significant at alpha level = 0.05. Salinity = soil salinity, Clay = soil clay content, STN = soil total nitrogen, STP = soil total phosphorus, avaP = soil available phosphorus.**

| Abundant fungi | | | | | |
| --- | --- | --- | --- | --- | --- |
| Marginal *R*^2^= 0.103 | | Conditional *R*^2^= 0.460 | |  |  |
|  | Estimate | *t* | *Pr*(>|*t*|) | Chisq | *Pr*(>Chisq) |
| Salinity | 0.120 | 1.891 | 0.060 | 3.577 | 0.059 |
| pH | 0.118 | 1.814 | 0.072 | 3.291 | 0.070 |
| Clay | **0.121** | **2.312** | **0.022** | **5.347** | **0.021** |
| STN | -0.036 | -0.629 | 0.530 | 0.396 | 0.529 |
| STP | **-0.134** | **-2.140** | **0.034** | **4.580** | **0.032** |
| avaP | -0.095 | -1.875 | 0.063 | 3.515 | 0.061 |
|  |  |  |  |  |  |
| Rare fungi | | | | | |
| Marginal *R*^2^= 0.099 | | Conditional *R*^2^= 0.270 | |  |  |
|  | Estimate | *t* | *Pr*(>|*t*|) | Chisq | *Pr*(>Chisq) |
| Salinity | 0.132 | 1.671 | 0.097 | 2.792 | 0.095 |
| pH | **0.201** | **2.564** | **0.012** | **6.574** | **0.010** |
| Clay | 0.103 | 1.609 | 0.110 | 2.588 | 0.108 |
| STN | 0.037 | 0.535 | 0.593 | 0.286 | 0.592 |
| STP | -0.073 | -0.956 | 0.341 | 0.914 | 0.339 |
| avaP | **-0.133** | **-2.128** | **0.035** | **4.528** | **0.033** |

**Supplemental Table 15.** Db-RDA results of the rare and abundant soil fungal guilds. Abundant, OTUs relative abundance above 0.01%; Rare, OTUs relative abundance below 0.01%. *SS*, sum of squares. Values in bold are significant at alpha level = 0.05. GD = geographic distance, Salinity = soil salinity, Clay = soil clay content, STN = soil total nitrogen, STP = soil total phosphorus, avaP = soil available phosphorus.

| Variable | Saprotrophic fungi | | | | Pathotrophic fungi | | | | Symbiotrophic fungi | | | |
| --- | --- | --- | --- | --- | --- | --- | --- | --- | --- | --- | --- | --- |
|  | Abundant | | Rare | | Abundant | | Rare | | Abundant | | Rare | |
|  | *SS* | *P* | *SS* | *P* | *SS* | *P* | *SS* | *P* | *SS* | *P* | *SS* | *P* |
| GD | **5.079** | **0.001** | **2.680** | **0.001** | **7.162** | **0.001** | **2.416** | **0.001** | **3.933** | **0.001** | **2.702** | **0.001** |
| Salinity | 1.079 | 0.055 | **1.383** | **0.001** | **1.116** | **0.015** | **1.222** | **0.005** | 0.363 | 0.544 | 0.956 | 0.275 |
| pH | **1.647** | **0.005** | **1.184** | **0.019** | 0.605 | 0.303 | 1.121 | 0.057 | 0.513 | 0.327 | 0.890 | 0.385 |
| Clay | **1.744** | **0.004** | **1.208** | **0.015** | **1.128** | **0.009** | **1.217** | **0.012** | 0.866 | 0.107 | 1.038 | 0.150 |
| STN | **2.522** | **0.001** | **1.584** | **0.001** | **1.720** | **0.001** | **1.293** | **0.005** | 0.477 | 0.419 | **1.314** | **0.015** |
| STP | 0.997 | 0.065 | **1.322** | **0.004** | **1.008** | **0.019** | **1.272** | **0.004** | **2.120** | **0.002** | 1.024 | 0.155 |
| avaP | **1.565** | **0.003** | **1.172** | **0.019** | **1.132** | **0.016** | **1.213** | **0.012** | 0.420 | 0.479 | 1.137 | 0.077 |
| Depth | **1.422** | **0.016** | **1.274** | **0.006** | 0.836 | 0.081 | **1.542** | **0.001** | 0.825 | 0.120 | **1.237** | **0.033** |
| Residual | 102.709 |  | 158.027 |  | 93.172 |  | 157.169 |  | 81.300 |  | 146.546 |  |

**Supplemental Table 16.** Summary of the linear mixed models for the responses of alkaline phosphatase to functional fungal diversity. Soil depth and sampling site were considered as random factors. Values in bold are significant at alpha level = 0.05. ALP = alkaline phosphatase.

| Diversity | | Model=lme(ALP~ diversity, random= ~1|site ~1|depth ) | | | |
| --- | --- | --- | --- | --- | --- |
|  |  | Slope | *df* | *t* | *Pr*(>\|*t*\|) |
| Saprotrophic fungi | Species richness | -2.298 | 176 | **-3.087** | **0.002** |
|  | Exponential of Shannon entropy | -0.480 | 177 | -0.648 | 0.518 |
|  | Inverse Simpson | -0.884 | 175 | -1.214 | 0.226 |
| Pathotrophic fungi | Species richness | -2.316 | 178 | **-3.170** | **0.002** |
|  | Exponential of Shannon entropy | -0.734 | 166 | -1.018 | 0.310 |
|  | Inverse Simpson | -0.613 | 170 | -0.852 | 0.396 |
| Symbiotrophic fungi | Species richness | -1.525 | 164 | -1.908 | 0.058 |
|  | Exponential of Shannon entropy | -1.282 | 174 | -1.693 | 0.092 |
|  | Inverse Simpson | -0.995 | 172 | -1.324 | 0.187 |


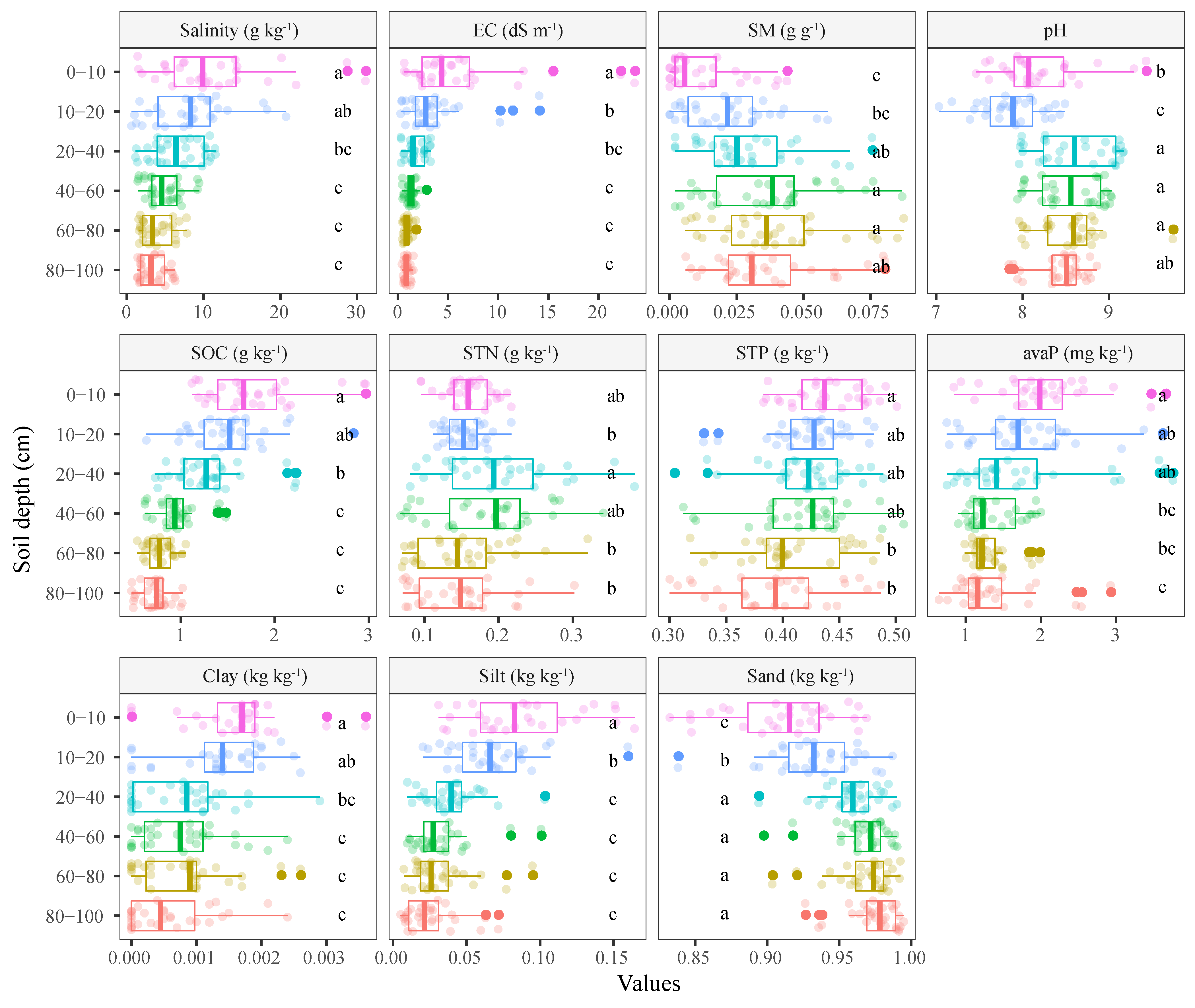


**Supplemental Figure 1**. Vertical distributions of soil properties. Different letters denote statistically significant differences. Salinity = soil salinity, EC = soil electronic conductivity, SM = soil moisture, SOC = soil organic carbon, STN = soil total nitrogen, STP = soil total phosphorus, avaP = available phosphorus, Clay = soil clay content, Silt = soil silt content, Sand = soil sand content.





**Supplemental Figure 2**. Geographic distribution of aboveground plant biomass along the gradient of geographic distance. The line is derived from the general linear models, and the dash line denotes insignificant regression coefficients.


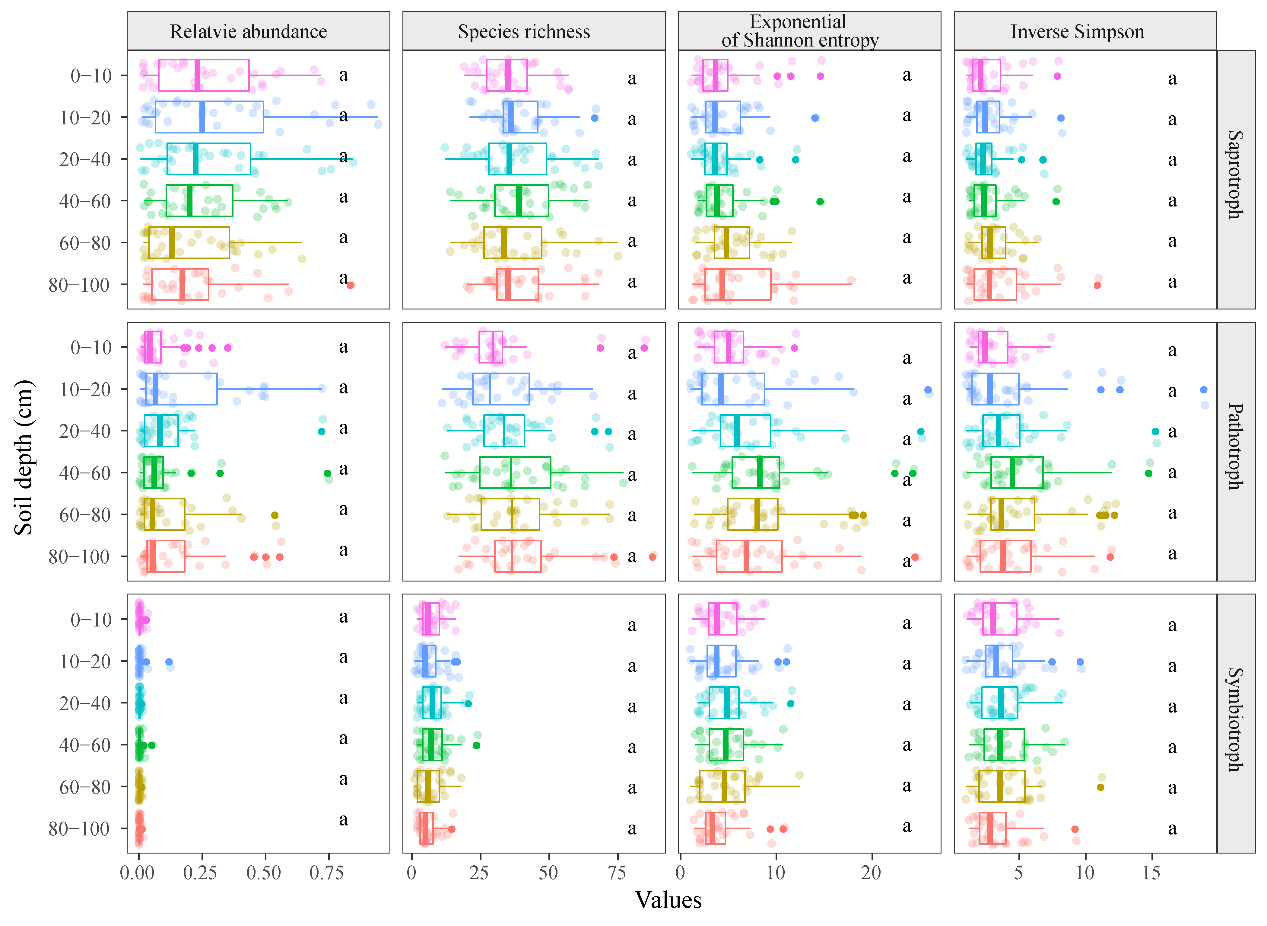


**Supplemental Figure 3**. Vertical distributions of the relative abundance and alpha diversity of functional guilds of soil fungi. Different letters denote statistically significant differences.


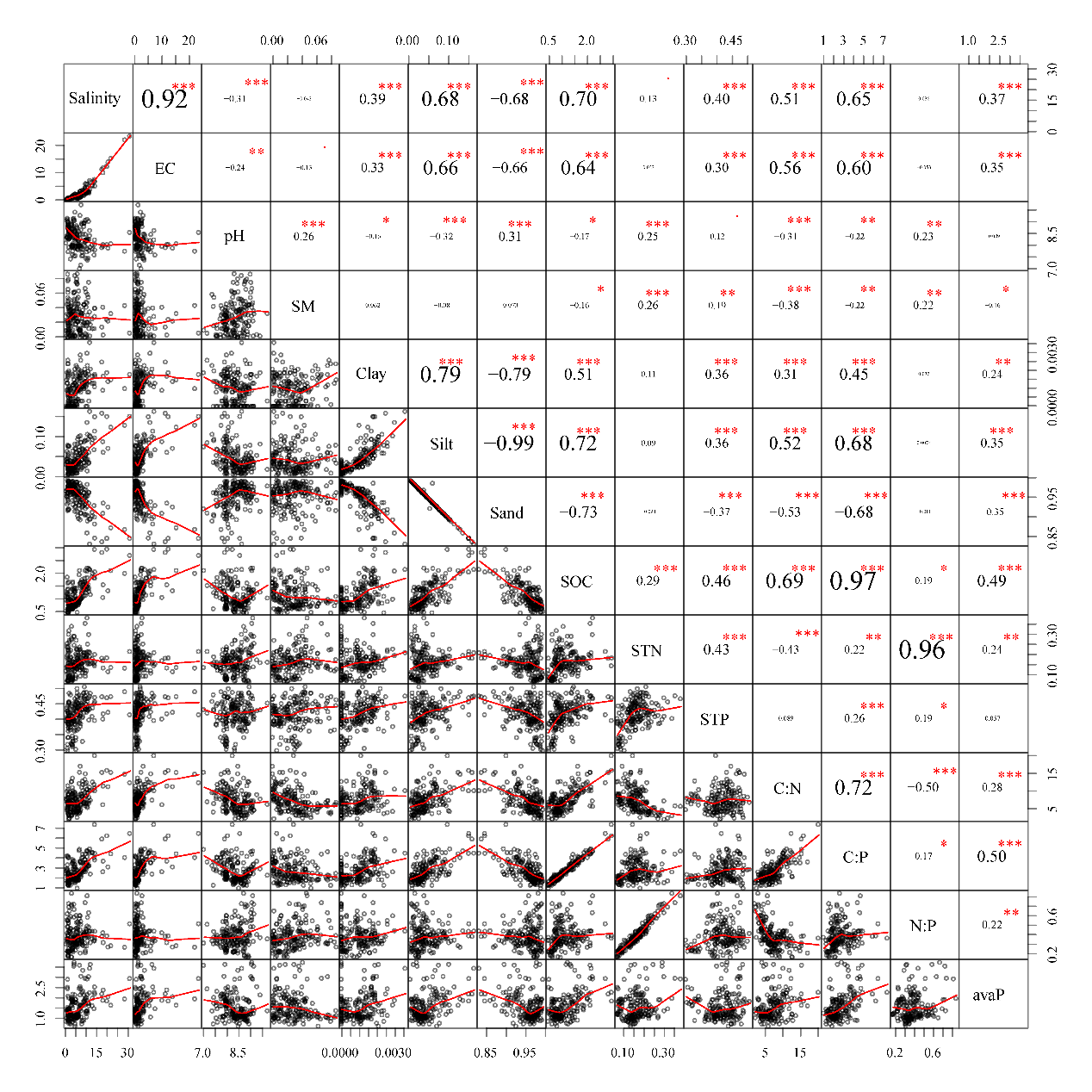


**Supplemental Figure 4**. Pearson correlations among soil properties. Values in the upper triangle are the correlation coefficients. Salinity, soil salinity (g kg^-1^); EC, soil electronic conductivity (dS m^-1^); SM, soil moisture (g g^-1^); Clay, soil clay content (%); Silt, soil silt content (%); Sand, soil sand content (%); SOC, soil organic carbon (g kg^-1^); STN, soil total nitrogen (g kg^-1^); STP, soil total phosphorus (g kg^-1^); C:N, ratio of SOC to STN; C:P, ratio of SOC to STP; N:P, ratio of STN to STP; avaP, soil available phosphorus (mg kg^-1^). ***, *P*<0.001; **, *P*<0.01; *, *P*<0.05.


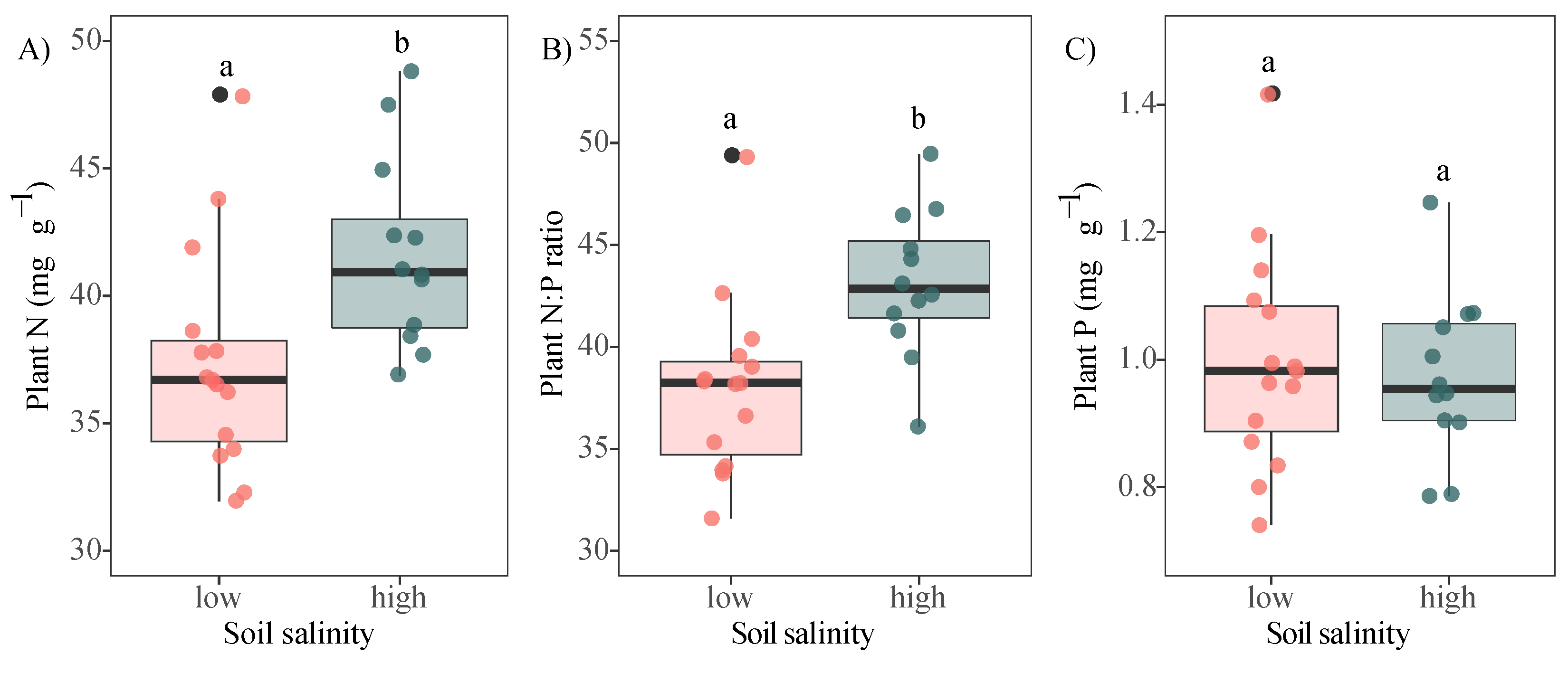


**Supplemental Figure 5**. Plant leaf nitrogen, phosphorus, and the ratio of nitrogen to phosphorus at the sites of high and low salinity in the shelterbelts. High salinity: S01, S04, S07, S10, and S13 sites; Low salinity: S16, S19, S23, S28, and S34 sites. (A), plant nitrogen; (B), plant nitrogen to phosphorus ratio; (C), plant phosphorus.

Different letters denote statistically significant differences.


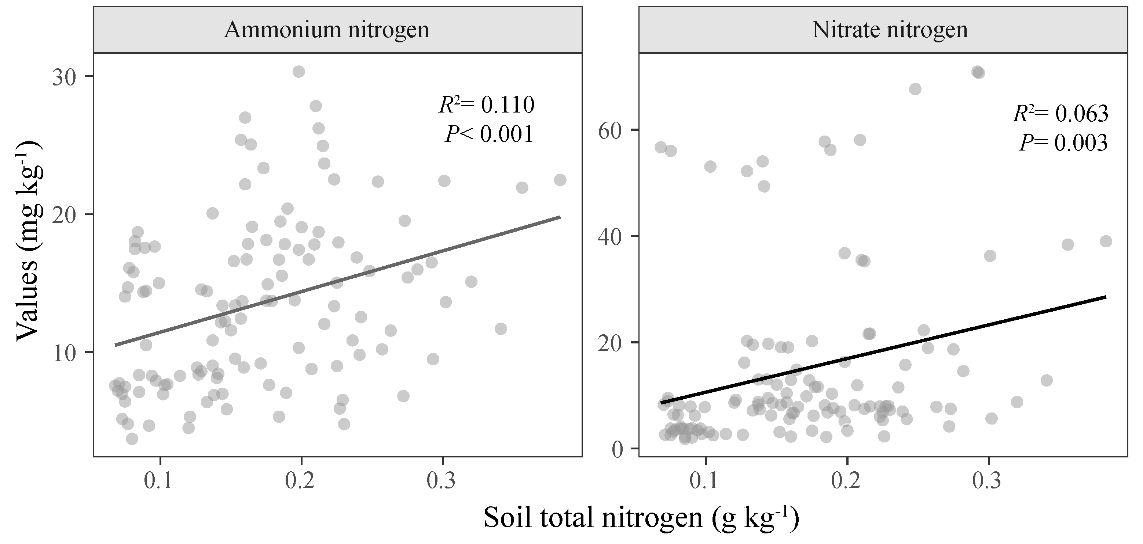


**Supplemental Figure 6**. Correlations of soil ammonium and nitrate nitrogen with soil total nitrogen. Lines are derived from the general linear models, and solid lines denote significant regression coefficients.
